# Mutations in feline infectious peritonitis virus nonstructural protein 14/16 methyltransferase attenuate the pathogenicity of the virus in cats

**DOI:** 10.1128/jvi.00839-25

**Published:** 2025-09-09

**Authors:** Zhe Jiao, Jia Li, Pengpeng Wang, Yuanyuan Yan, Lingying Fang, Yuanyuan Chen, Xiaoshuai Hu, Yuejun Shi, Guiqing Peng

**Affiliations:** 1 National Key Laboratory of Agricultural Microbiology, College of Veterinary Medicine, Huazhong Agricultural University 627716, Wuhan, China; 2 Key Laboratory of Preventive Veterinary Medicine in Hubei Province, The Cooperative Innovation Center for Sustainable Pig Production, Wuhan, China; 3 Hongshan Lab, Wuhan, China; Loyola University Chicago - Health Sciences Campus, Maywood, Illinois, USA

**Keywords:** feline infectious peritonitis virus, nsp14, N7-methyltransferase, nsp16, 2’-O-methyltransferase, live attenuated vaccine

## Abstract

Feline infectious peritonitis virus (FIPV) can cause an immune-mediated disease that is fatal to felines, but there is a lack of clinically effective protection conferred by vaccines. The methyltransferase (MTase) activity of the coronavirus nonstructural proteins nsp14 and nsp16 affects virulence, but there are no studies on the effect of nsp14 and nsp16 mutations affecting enzyme activity on the virulence of FIPV. In this study, we successfully rescued two mutant strains based on the previous infectious clone QS-79, named FIPV QS-79 dnsp14 and dnsp16, by mutating the MTase active sites of nsp14 (N415) and nsp16 (D129). The dnsp14 and dnsp16 exhibited similar syncytium formation ability and growth kinetics as their parental strains *in vitro*. Moreover, both mutants stimulated increased expression of interferon and cytokines in a macrophage lineage cell line. The dnsp14 and dnsp16 exhibited significantly lower pathogenicity in cats, with a 75% reduction in mortality compared to the wild type. The dnsp14 and dnsp16 were administered to cats at high (10^5^ TCID_50_) and low (10^4^ TCID_50_) doses for immunization purposes. The dnsp14 resulted in the production of high neutralizing antibody titers (>1:156) at both doses, whereas the dnsp16 only induced low neutralizing antibody titers (<1:64). And the dnsp14 provided protection to 50% of the cats at both immunization doses, whereas the dnsp16 failed to provide protection to cats of the challenge assays. The above results show that these two mutant strains are less pathogenic in cats, and dnsp14 induces a better humoral immune response and protection than dnsp16.

**IMPORTANCE:**

Feline infectious peritonitis virus is a significant pathogen that affects felines worldwide, and its high fatality rate has long been a concern in the pet medical industry. Previous studies have indicated that the virus encodes nsp14 and nsp16 methyltransferases, which play a crucial role in viral genome replication and evasion of innate immunity. In this study, we aimed to inhibit the MTase activity by mutating the methyltransferase of feline infectious peritonitis virus nonstructural protein 14/16. This mutation resulted in the construction of a vaccine candidate with reduced virulence, efficient replication in the host, and the ability to provide partial protection from the virulent parental virus. Our findings offer valuable insights for the development of live attenuated vaccines that target the nsp14/nsp16 MTase of feline coronaviruses.

## INTRODUCTION

Feline coronavirus (FCoV) is a very common pathogen in domestic and wild cat populations and is divided into feline infectious peritonitis virus (FIPV) and feline enteric coronavirus (FECV) according to pathogenicity (1, 2). FIPV is the cause of a highly fatal disease in cats called feline infectious peritonitis (FIP) (3). FIP can occur in cats of all ages and breeds and can be 100% fatal once it develops, and there are currently no approved drugs or effective vaccines (4–7). The virological classification of FIPV indicates that it is a member of Nidovirales, Coronaviridae, Orthocoronavirinae, and the alphacoronavirus genus (8). Its genome is a single-stranded positive-stranded RNA of approximately 29 kb in size, and the genome exhibits a gene sequence similar to that of other coronaviruses (9, 10). The open reading frame ORF-1 following the 5′ end cap structure accounts for two-thirds of the entire genome, and the sequence encodes viral replicase-associated polyproteins, including pp1a and pp1ab, that, after translation, are usually processed to form 16 nonstructural proteins that form replication-transcription complexes, thereby completing coronavirus genome replication, transcription (11, 12). The remaining one-third of the sequence at the 3′ end encodes the S, E, N, and M proteins that make up the viral particle and ORF 3abc and ORF 7ab that encode accessory proteins of unknown function (13, 14).

The capping of coronavirus mRNA is critical for viral replication, transcription, translation, and evasion of the host immune response. Previous studies have shown that a variety of nonstructural proteins play a role in the cap addition process, including nsp14 and nsp16, which are involved in mRNA cap methylation, a process that is critical for the formation of the cap-0 and cap-1 structures (15). Mutants of coronavirus nsp14 and nsp16 are effective platforms for the design of live attenuated vaccines. It is worth noting that the nsp14 subunit replicase contains 3′-to-5′ exoribonuclease (ExoN) and guanine-N7-methyltransferase domains (16). In mouse hepatitis virus (MHV) and severe acute respiratory syndrome coronavirus (SARS-CoV), deletion or mutation of the conserved DEDD motif within the nsp14 exonuclease structural domain results in reduced or complete loss of viral replication and leads to reduced viral replication fidelity (17). Transmissible gastroenteritis virus (TGEV) lacking ExoN activity cannot proliferate in cells, but specific mutations within zinc finger 1 (ZF-C) in the ExoN structural domain result in recombinant viruses with growth kinetics that do not differ from those of the parental strain (16). The mutation of the nsp14 S-adenosyl methionine (SAM)-binding motif to DxG in porcine epidemic diarrhea virus (PEDV) completely eliminates the N7-MTase activity of nsp14, resulting in the inability of PEDV to replicate. However, the recombinant strain rPEDV-D350A with a single mutation (D350A) in nsp14 retained 29.0% N7-MTase activity, showed replication and gene expression defects in cell culture, and induced significantly higher type I and type III interferon responses than the wild type (18). Menachery successfully rescued SARS-CoV recombinant viruses with K46A, K170A, and D130A mutations in the nsp16 catalytic quadruplex in cells, and these SARS-CoV recombinant mutants exhibited enhanced type I IFN sensitivity similar to that of MHV and HCoV-229E mutants (19, 20). Mutations in the nsp16 enzyme active site in SARS-CoV, severe acute respiratory syndrome coronavirus 2 (SARS-CoV-2), and Middle East respiratory syndrome coronavirus (MERS-CoV) result in the induction of high levels of neutralizing antibodies *in vivo*, providing protection to subject animals from disease following exposure to lethal viral challenge (21–23). Using the infectious cDNA clone of a virulent PEDV strain as the backbone, researchers constructed a recombinant virus with defective nsp16 2′-O-MTase activity and S endocytosis signaling. Recombinant mutants replicated less efficiently *in vitro* than the wild type but induced stronger type I and III interferon responses. The pathogenicity and immunogenicity of the mutants were evaluated, and it was found that the mutant virus was completely attenuated and provided 100% immune protection to piglets (24).

Although the design of the FIPV vaccine has been ongoing for decades, the production of a safe and effective vaccine remains a challenge for academic and commercial researchers. One reason why the development of FIPV vaccines is currently hindered is that when FIPV vaccines induce weaker cellular immunity, the dominance of humoral immunity may increase the risk of FIP (25). Researchers immunized kittens with a recombinant cowpox virus expressing the FCoV S protein, and although the vaccine was able to induce an S-specific antibody response and the production of low levels of neutralizing antibodies, it resulted in post-challenge susceptibility in kittens (26). Therefore, live attenuated vaccines are ideal for preventing FIPV infection, as the nonlethal virus persists in the body in a subclinical state, allowing the body to remain immune and thus prevent infection (27). The MTase activities of nsp14 and nsp16 are highly conserved in several coronaviruses, so for FIPV, loss of the MTase activities of nsp14 and nsp16 could be a strategy for virulence attenuation and vaccine design.

QS-79 is an infectious clone previously constructed in our laboratory, which contains the entire genome of FIPV QS strain except the spike protein. We used the 791146 spike protein to replace the original spike protein to improve the cell adaptability of the strain and confirmed that the strain is infectious with high pathogenicity (28). In this study, the infectious cDNA clone of QS-79 was used as a backbone to mutate the enzyme active sites of nsp14 and nsp16, and the effects of the mutant virus growth kinetics and enzyme activity abrogation on host innate immunity were investigated at the cellular level to further assess the pathogenicity and immunogenicity of the mutant virus at the animal level. These studies can help us better understand the effect of MTase on coronavirus replication and provide an important reference for FIPV vaccine design.

## RESULTS

### FIPV nsp14 N415 and nsp16 D129 are critical MTase active sites

**Fig 1:**
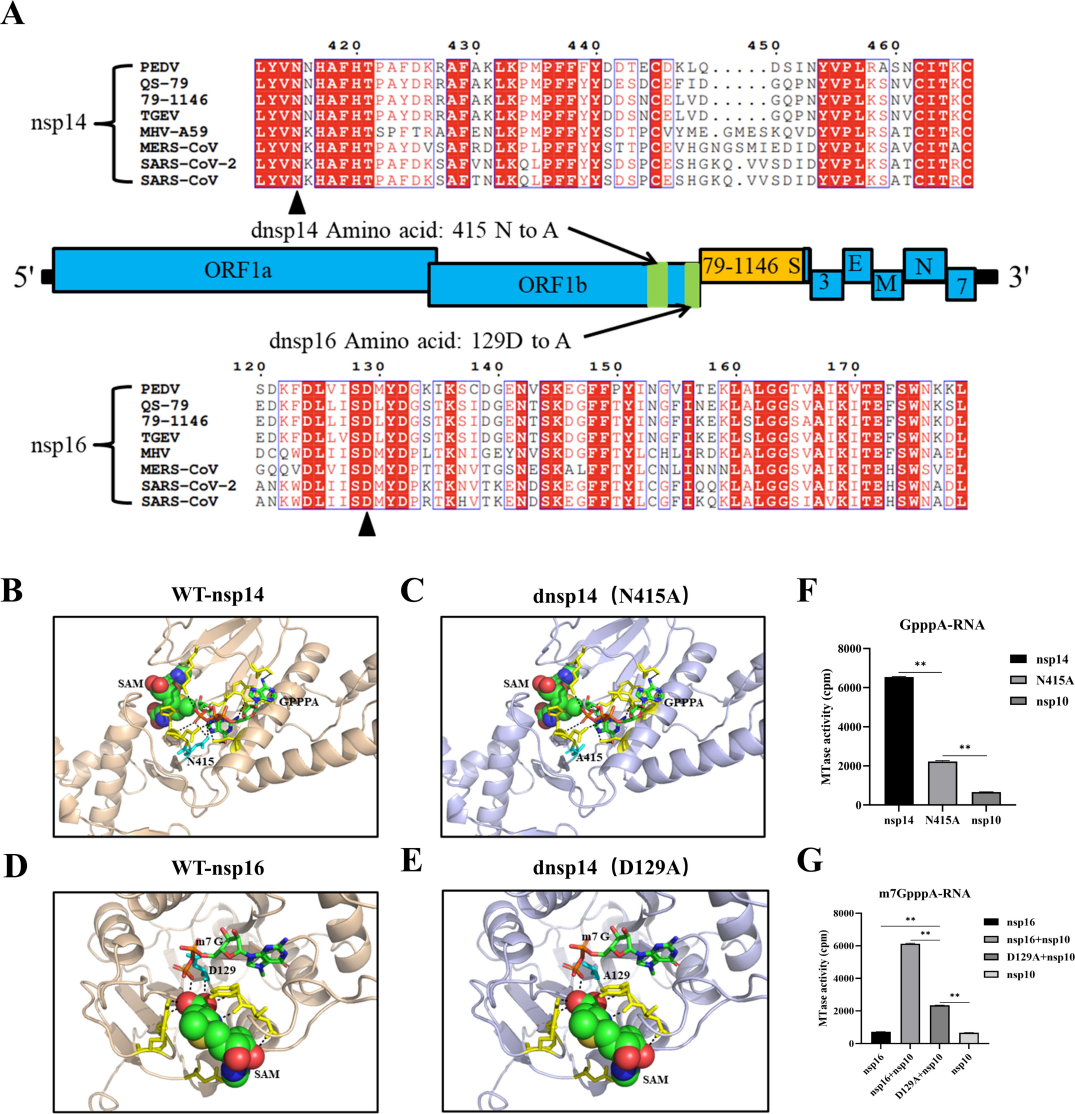
FIPV nsp14 N415 and nsp16 D129 are MTase critical active sites. (**A**) Amino acid sequence comparison of the active site of coronavirus nsp14/nsp16 methyltransferase. Asparagine (N) at nsp14 415 and aspartic acid (**D**) at nsp16 129 are mutant sites (shown in black triangles), which are highly conserved in many coronaviruses. (**B, C**) Prediction of wild-type and mutant nsp14 (dnsp14) methyltransferases binding pockets to SAM. When the amino acid at position 415 is mutated to alanine, the hydrogen bond formation of the oxygen atom on the NSP14 415 site and the guanosine phosphate group is destroyed. (**D, E**) Prediction of wild-type and mutant nsp16 (dnsp16) methyltransferases binding pockets to SAM. When the amino acid at position 129 mutates to alanine, it destroys the hydrogen bond formation between the nsp16 129 site and the oxygen atom on the SAM, which affects the stability and positioning of the SAM closer to m7G. (**F, G**) MTase activity of nsp14, nsp16, nsp14 N415A mutation, and nsp16 D129A mutation. Nsp16 requires the cofactor nsp10 for MTase activity; thus, nsp10 was added as a control.

Based on previous studies, it has been found that a complete knockdown of MTase activity of nsp14 or nsp16 would result in the virus being unable to be rescued. To develop a strategy to obtain a recombinant mutant FIPV that can replicate in cells, we analyzed FIPV 79-1146, PEDV CV777, MHV A59, TGEV WH-1, SARS-CoV-2 Wuhan-Hu-1, SARS-CoV Tor2, and MERS-CoV EMC by sequence alignment. The MTase active sites of nsp14 and nsp16 were found to be highly conserved in multiple coronaviruses, and asparagine at nsp14 position 415 and aspartic acid at nsp16 position 129 were finally identified as mutation site targets (Fig. 1A). Structural simulations of the FIPV QS-79 parental strain and dnsp14 and dnsp16 mutant strains were performed via the AlphaFold2 online website, and the SAM-binding pockets of the wild-type and mutant strains were predicted using AutoDock Vina. As shown in the figure, molecular docking showed that the side chain at the N415 site of nsp14 is involved in β-strand formation, and together with the neighboring α-helix, they form a pocket that stabilizes the cap nucleus structure (GPPPA) the SAM, facilitating the participation of nsp14 in the methyl transfer process of addition to the RNA cap (Fig. 1B). When the amino acid at position 415 is mutated to alanine, the formation of hydrogen bonds between the nsp14 415 site and the oxygen atom on the guanosine phosphate group is disrupted (Fig. 1C). Modeling results for nsp16 showed that the aspartic acid at position 129 is located closer to m7G (cap-0 structure) within the SAM-binding pocket, and this site and other amino acids within the pocket stabilize and position SAM toward m7G through intermolecular interactions (Fig. 1D and E). To verify the effect of these two sites on enzyme activity, we expressed FIPV QS-79 nsp16, nsp14, nsp10, nsp14 N415A site mutation, and nsp16 D129A site mutation protein, then measured the MTase activity using the corresponding substrate. The results showed that the methylase activity of nsp14 N415 and nsp16 D129 mutations was greatly reduced (Fig. 1F). These results demonstrate that the nsp14 N415 site and nsp16 D129 site are critical MTase active sites; meanwhile, nsp14 and nsp16 are involved in FIPV QS-79 RNA capping methylation.

### Rescue of mutant FIPV strains dnsp14 and dnsp16

**Fig 2:**
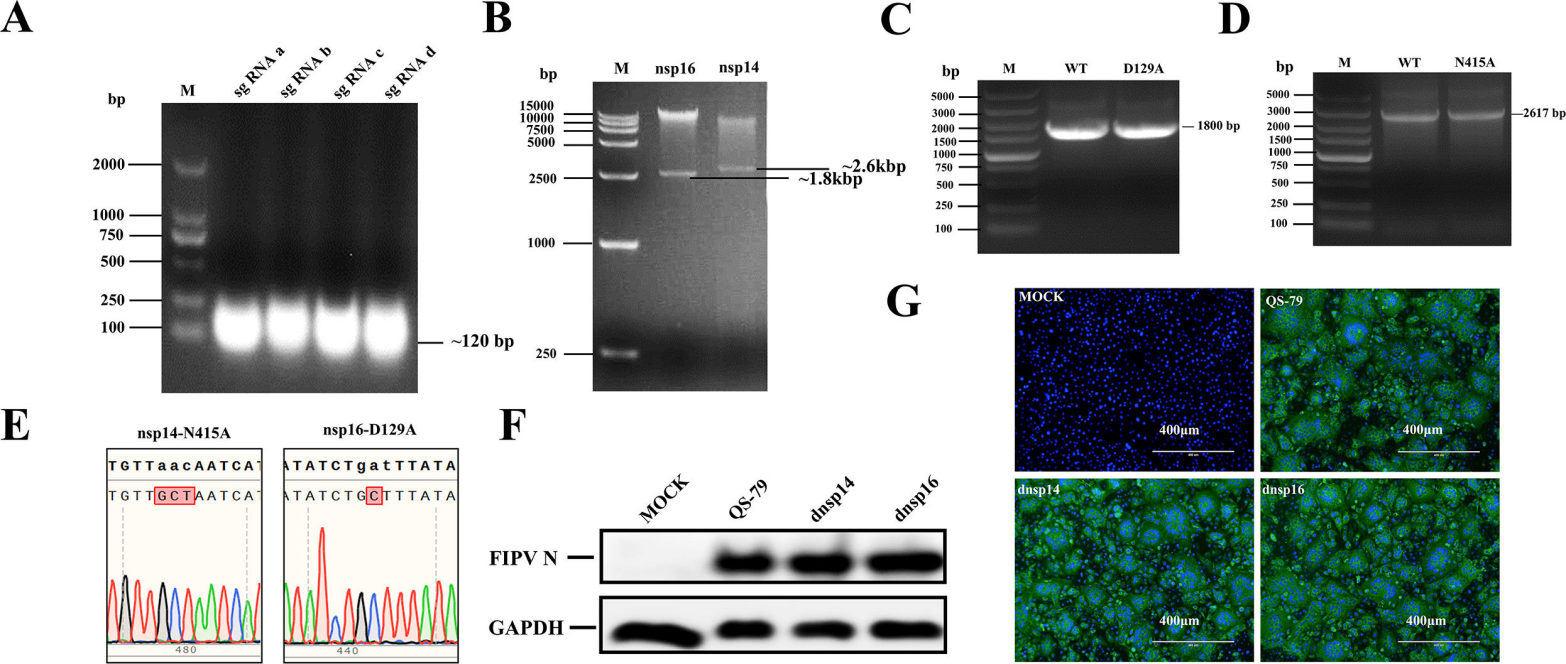
Rescue of mutant FIPV strains dnsp14 and dnsp16. (**A**) Identification of sgRNA transcribed *in vitro* by agarose gel electrophoresis, fragment size of about 120 bp. (**B**) The BAC QS 79 clones digested by Cas9 guided by sgRNA were detected by electrophoresis. (**C, D**) Two gene fragments with nsp14 N415A and nsp16 D129A locus mutation obtained by fusion PCR. (**E**) Site analysis of nsp14-N415A and nsp16-D129A by sequencing. (**F, G**) Recovery of the mutant FIPV strains dnsp14 and dnsp16. Antigen signals identified by WB and IFA were observed at 36 h post-transfection.

The viral genome was manipulated by *in vitro* CRISPR‒Cas9 technology to design recombinant mutants with abrogation of QS-79 nsp14 and QS-79 nsp16 enzymatic activities. First, we performed sgRNA *in vitro* transcription and agarose gel electrophoresis identification. The fragment size was correct, the integrity and purity were confirmed (Fig. 2A). The QS-79 BAC plasmid was digested using Cas9 enzyme and sgRNA, successfully producing a linearized vector and a fragment containing the gene of interest (Fig. 2B). Point mutation by fusion PCR successfully produced two gene fragments, one for the nsp14 N415A site mutation and one for the nsp16 D129A site mutation (Fig. 2C and D). Mutation fragments and BAC plasmids were recombinantly combined by insertion via transformation, and the resultant plasmids were sequenced. The sequencing results showed that the 415 position of nsp14 and the 129 position of nsp16 were mutated to encode alanine, indicating that dnsp14 BAC and dnsp16 BAC were successfully constructed (Fig. 2E). The dnsp14 BAC, dnsp16 BAC, and the positive control QS-79 BAC were inserted into HEK-293T cells via transfection, and after 24 h, CRFK cells were infected with cell supernatant for 36 h. Western blot and indirect immunofluorescence experiments were performed, and FIPV virus N protein was successfully detected (Fig. 2F and G), proving that the dnsp14 and dnsp16 mutant strains had been successfully rescued in CRFK cells.

### dnsp14 and dnsp16 do not exhibit diminished proliferation *in vitro* but induce enhanced immune responses

**Fig 3:**
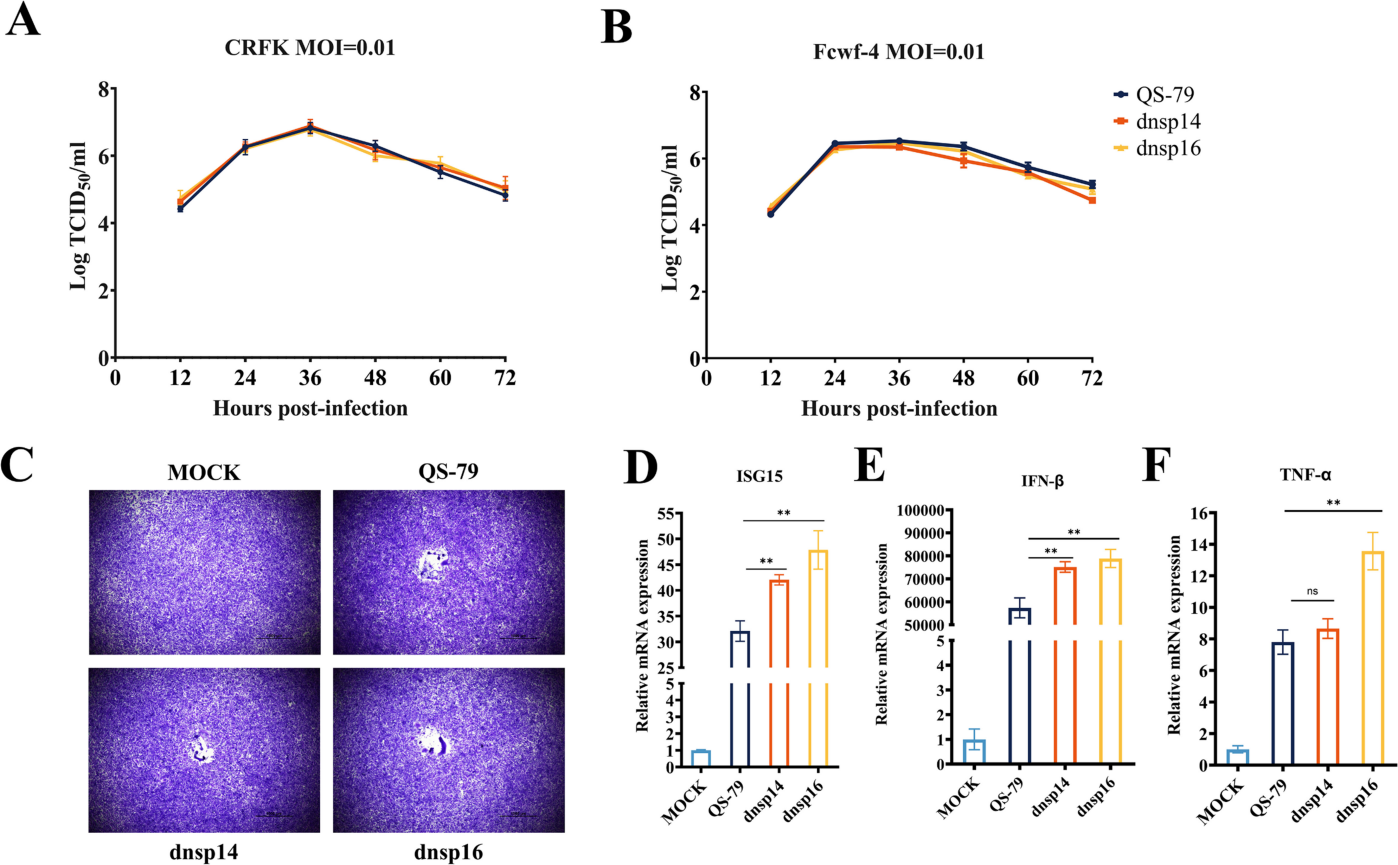
The dnsp14 and dnsp16 do not diminish proliferation *in vitro* but induce stronger immune responses. (**A, B**) Growth kinetics of QS-79, dnsp14, and dnsp16 on CRFK and Fcwf-4 cells. The growth kinetic curves showed that there were no significant differences in the proliferation trend of mutant and parental strains after infection with the two cell lines, and there were no significant differences in viral titer and peak viral titer at each time point. (**C**) Viral etiolation assays of QS-79, dnsp14, and dnsp16 on Fcwf-4 cells. No significant difference in the etiolation formation ability between the mutant and parental strain infections after crystalline violet staining. (**D-F**) Changes of ISG15, IFN-β, and TNF-α in Fcwf-4 cells after virus infection. The dnsp14 and dnsp16 mutant strains induce higher levels of ISG15 and IFN-β expression. TNF-α expression in cells infected with the dnsp16 mutant strain is elevated. Error bars represent the standard error of the mean (SEM). Statistical significance compared to the QS-79 strain at each concentration was determined by one-way ANOVA and indicated with an asterisk (*); **P < 0.01.

To characterize the biological properties of the mutant strains dnsp14 and dnsp16 *in vitro*, we infected CRFK and Fcwf-4 cells with dnsp14, dnsp16, and QS-79 at a multiplicity of infection (MOI) of 0.01, and we collected viral cell supernatants every 12 h to perform a TCID_50_ assay. The growth kinetic curves indicated that there was no significant difference in the proliferation, viral titer, or peak viral titer at each time point between the mutant and parental strains after infection of the two cell lines (Fig. 3A and 2B). Viral syncytium formation assays were also performed with Fcwf-4 cells and, after crystal violet staining, showed no significant difference in the syncytium formation ability between the mutant and parental strain infections (Fig. 3C). Fcwf-4 cells were infected at a dose of an MOI of 0.01 and incubated for a total of 24 h, and RNA from the cells was extracted for real-time PCR assays. The results showed that dnsp14 and dnsp16 induced significantly higher levels of mRNA expression of IFN-β and ISG15 (Fig. 3D and E). In addition, it was observed that the expression of TNF-α in cells infected with the dnsp16 mutant strain was significantly increased, while there was no significant difference in the expression level of TNF-α after infection between the dnsp14 mutant strain and the QS-79 parental strain (Fig. 3F). These results show that the QS-79 nsp14 N415A site mutation and nsp16 D129A site mutation do not affect the proliferative ability of the virus *in vitro*, but the recombinant viruses can induce a stronger immune response.

### The pathogenicity of dnsp14 and dnsp16 is attenuated *in vivo*

**Fig 4:**
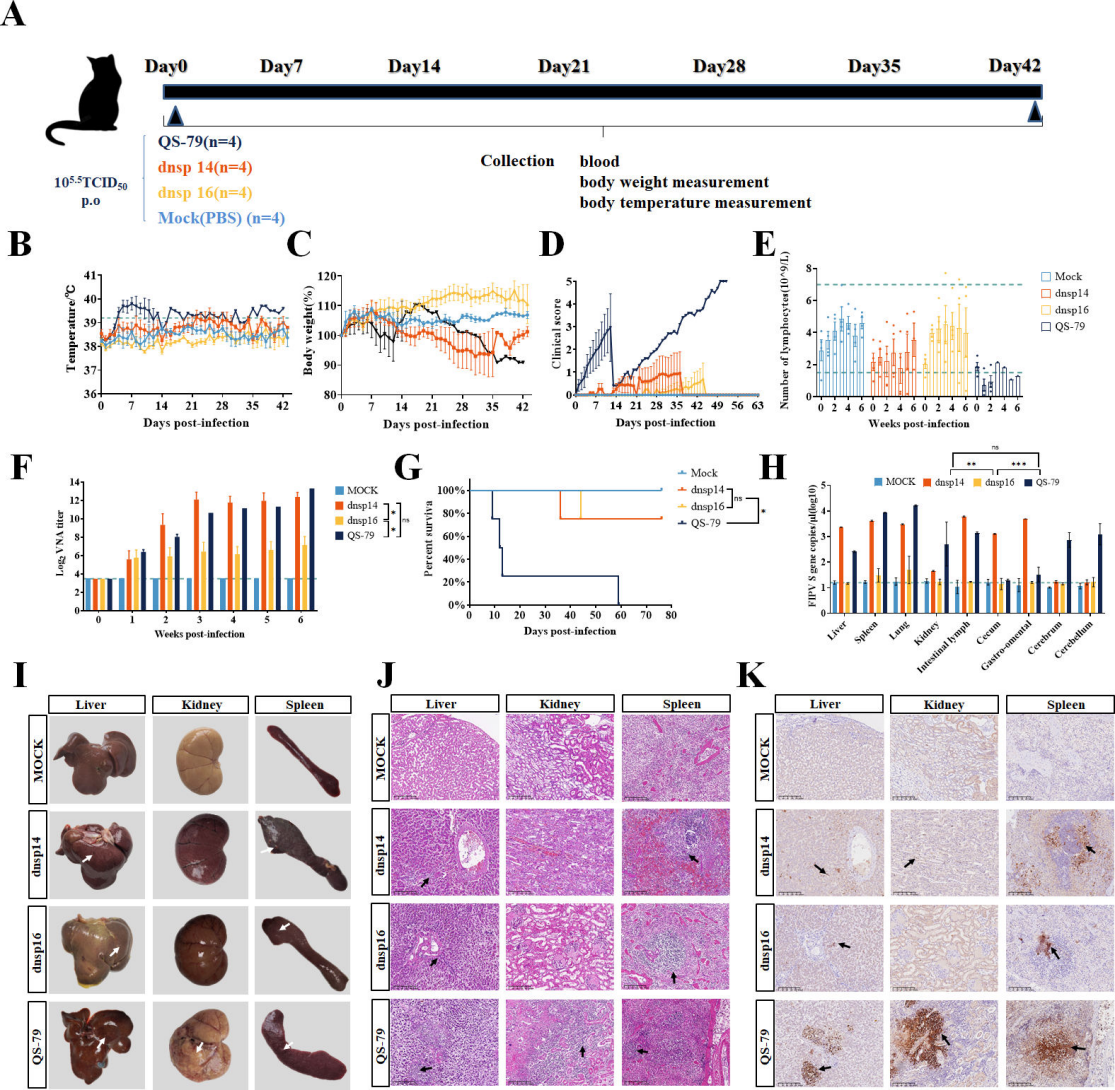
Evaluating the pathogenicity of dnsp14 and dnsp16 in cats. (**A**) Oral infection model timeline and grouping. (**B-E**) Changes in body temperature, body weight, clinical score, and lymphocyte count of infected cats. Cats with abnormal clinical status as follows: (A) cats with fever (>39°C); (**B**) cats with anorexia; (**C**) cats with lethargy; (**D**) cats with eye and nose discharge; and (**E**) cats with abnormal excretion. Scoring is based on the accumulation of the above-appearing symptoms, one point for each item. (**F**) Changes in neutralizing antibody titers after infection with each group. Infection with dnsp14 and QS-79 in adult cats can induce high levels of neutralizing antibodies. DNSP16 infection-induced antibody levels were slightly lower than in both groups. (**G**) Percent survival of the different cats in each group. The survival rate of cats in the QS-79 group was 0, while the survival rate of cats in both the dnsp14 and dnsp16 groups was 75%. (**H**) Viral load in each organ in the dead cats. There was no significant difference in viral load detection between the dnsp14 group and the QS-79 group, but both were significantly higher than those in the dnsp16 group. (**I**) Necropsy analysis of the dead cats. FIP typical fibrinous serositis or noncaseating granulomas (white arrows) were observed in the liver, kidneys, and spleen in the QS-79 group, while purulent granulomas in the dnsp14 and dnsp16 groups appeared in the liver and spleen with a lower degree of lesion than QS-79. (**J, K**) Histopathology analysis of the dead cats. In the analysis of H&E staining of the liver, kidneys, and spleen sections in the QS-79 group, granuloma-related lesions were surrounded by dense inflammatory infiltrates (black arrows). Immunohistochemical antirabbit antiserum against FIPV N protein (black arrows, brown) detected viral antigens in the macrophages in cat lesions. And the tissue damage in the dnsp14 and dnsp16 groups was less severe than in the QS-79 group. Error bars represent the standard error of the mean (SEM). Statistical significance compared to dnsp14, dnsp16, and QS-79 strain at each concentration was determined by one-way ANOVA and indicated with an asterisk (*); *P < 0.05, **P < 0.01, ***P < 0.001.

An oral challenge model in adult cats was used to evaluate the *in vivo* pathogenicity of the mutant strains (Fig. 4A). Sixteen healthy adult cats were randomly divided into four groups of four cats each; the cats in each of the three experimental groups received 1 mL of 10^5.5^ TCID_50_/mL dnsp14, dnsp16, and QS-79 orally, and the cats in the mock group received 1 mL of sterile PBS orally. Clinical scoring was performed based on appetite, mental status, ocular and nasal discharge, fecal excretion, and neurological signs. The experimental results showed that adult cats had increased body temperature and weight loss after infection with the QS-79 strain, while cats in the dnsp14- and dnsp16-infected groups did not have persistent fever during the entire trial period (Fig. 4B and C). The weight of the cats in the dnsp14 group decreased slightly after 1 week of infection and gradually recovered in the later stage. The weight of the cats in the dnsp16 group did not decrease, and the cats in the mock group did not have a temperature increase or weight loss. The clinical symptoms of adult cats in the QS-79 group continued to deteriorate after infection, manifested as depression, loss of appetite, inflammatory discharge in the eyes and nose, and decreased excretion. The dnsp14 group cats showed loss of appetite and depression along with weight loss, but these clinical manifestations disappeared with weight recovery, and their overall clinical score was significantly lower than that of the QS-79 infection group cats; meanwhile, the clinical symptoms of dnsp16 group cats were no different from those of the mock group cats, and no obvious clinical symptoms of FIP were found (Fig. 4D). Lymphocyte depletion is a typical feature of FIP, and routine blood test results showed that the number of lymphocytes in the QS-79-infected group dropped below the baseline level after 1 week, and the number of lymphocytes in the dnsp14- and dnsp16-infected groups and mock group fluctuated within the normal range throughout the trial period (Fig. 4E). The results of neutralizing antibody detection showed that infection of adult cats with QS-79, dnsp14, and dnsp16 could induce an increase in the level of neutralizing antibodies. There was no significant difference between the levels of antibodies induced by dnsp14 infection and QS-79 infection, the levels of antibodies induced by dnsp16 infection were lower than those in the other experimental two groups, and there was no neutralizing antibody production in the mock group (Fig. 4F). The expression of innate immune genes was examined, such as IFN-β, ISG15, and IFITM1, and the results showed that compared with the high expression after qs-79 infection, dnsp14, and dnsp16 infection reduced the expression of these genes (Fig. S1). The mortality results showed that all adult cats in the QS-79 group died within 9 weeks, three cats died within 2 weeks after infection, and the last cat that died had a longer course of disease, dying on day 59 after infection. One of the cats in the dnsp14 group died on the 36th day after the challenge, one of the cats in the dnsp16 group died on the 44th day after the challenge, and no cats in the mock group died (Fig. 4G). The dnsp14, dnsp16, QS-79, and MOCK groups were examined anatomically and pathologically on the 36th, 44th, 59th, and 59th days, respectively. According to anatomical and pathological examination, the gross lesions in the QS-79 group were obvious, with typical noncaseating granulomas of FIP observed in the liver, kidneys, and spleen, fibrinous plasmacytosis and purulent granulomas in the dnsp14 and dnsp16 groups, no obvious lesions in the kidneys, and no obvious lesions in the liver, kidneys, and spleen in the mock group (Fig. 4I). Hematoxylin and eosin (H&E) staining showed significant inflammatory cell infiltration and cell necrosis in the above lesions, but the lesions in the dnsp14 and dnsp16 groups were less severe than those in the QS-79 group, and the tissue from the mock group was morphologically intact without inflammatory infiltration (Fig. 4J). Immunohistochemical results showed that FIPV N protein was detected in the liver, spleen, and kidney of QS-79-infected cats, demonstrating that the organ lesions in QS-79-infected cats were caused by FIPV. Brown staining signals, indicating regions of pathogenesis, were also observed in the liver and spleen of dnsp14- and dnsp16-infected cats, with no significant positive signals in the kidney and no positive signals in any of the mock group cats (Fig. 4K). The assays of tissue organ viral loads showed that the viral load of the dnsp16 group was significantly lower than that of the QS-79 and dnsp14 groups, and viral particles were detected only in the spleen and lung (Fig. 4H). These results indicated that the two mutant strains were successfully attenuated *in vivo*.

### Lowering the immunization dose reduced mortality of dnsp16 immunization but did not affect dnsp14 immunization

**Fig 5:**
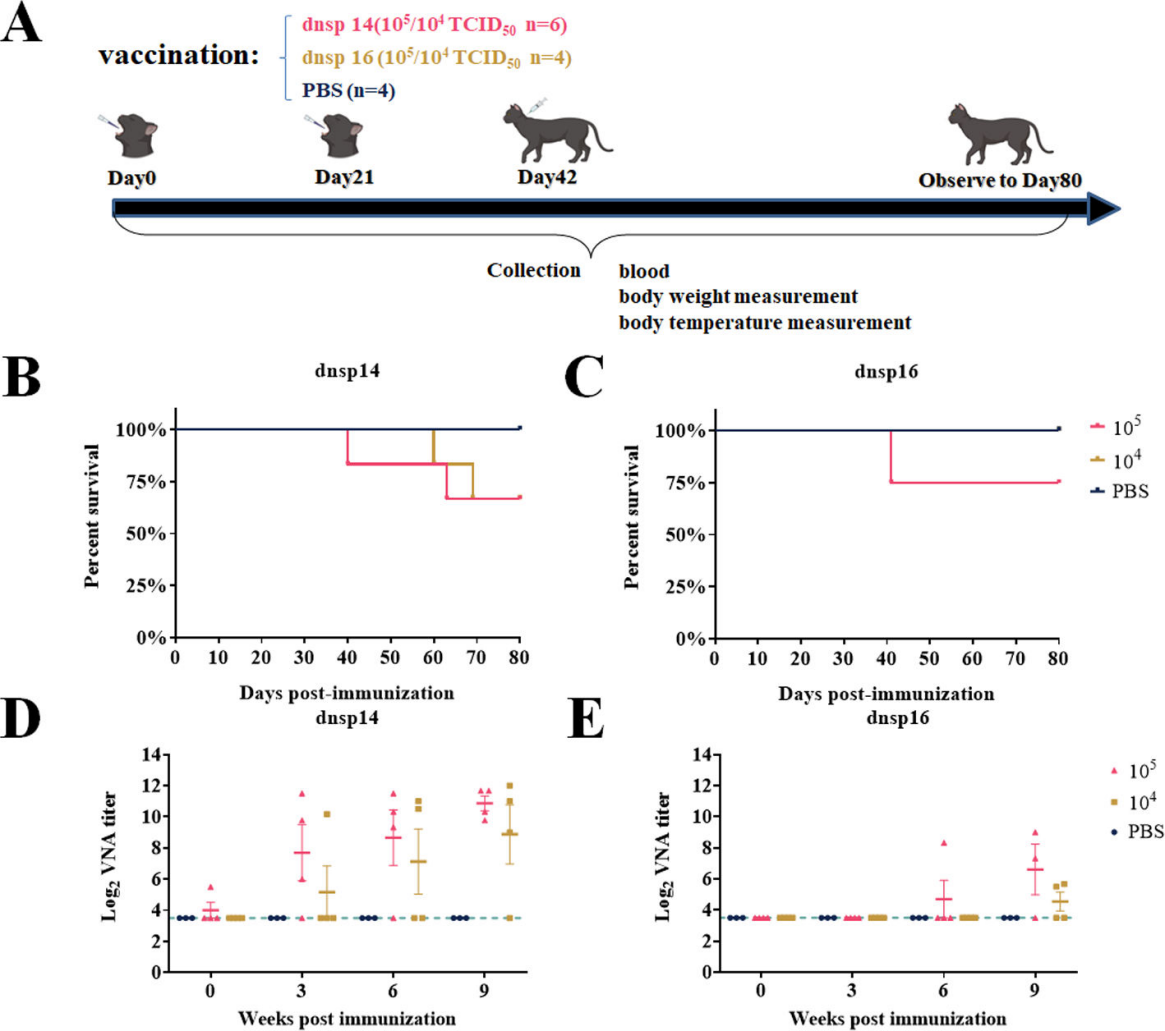
Lowering the immunization dose increases the safety of dnsp16 immunization, but does not increase dnsp14. (**A**) Immunization and challenge program for adult cats. For dnsp14 and dnsp16 mutant strains, high-dose (10^5^ TCID_50_) and low-dose (10^4^ TCID_50_) immunization groups were designed and immunized by two oral doses and one subcutaneous injection, each at 21-day intervals. The control group was immunized in the same manner with 1 mL of sterile PBS. (**B, C**) Survival rate after immunization with dnsp14 and dnsp16 mutant strains. The survival rate of dnsp14 in cats with both high-dose and low-dose immunization was 66%. The dnsp16 has a 25% mortality rate at high doses, while cats with low doses have a 100% survival rate. (**D, E**) Neutralizing antibody titers after immunization with dnsp14 and dnsp16 mutant strains. The dnsp14 mutant strain produced high levels of neutralizing antibodies after immunization, whereas the dnsp16 mutant strain produced only much lower levels of neutralizing antibodies than dnsp14 at both immunization doses.

The mutant strains still had a 25% mortality rate at a dose of 10^5.5^ TCID_50_, so the safety of immunization with the mutant strains was improved as much as possible by reducing the immunization dose. For the dnsp14 and dnsp16 mutant strains, high-dose (10^5^ TCID_50_) and low-dose (10^4^ TCID_50_) immunization groups were designed, and corresponding cats were immunized by two oral doses and one subcutaneous injection, each at 21-day intervals (Fig. 5A). The control group cats were immunized in the same manner with 1 mL of sterile PBS. The results showed that the mortality rate of dnsp14 was not dose dependent, with one-third of cats dying after both high- and low-dose immunizations (Fig. 5B). The dnsp14 mutant strain induced a neutralizing antibody titer of 1:1024 in all cats after three high-dose immunizations. After three immunizations at low doses, the immunized cats achieved an average antibody titer of more than 1:256, while one cat did not produce neutralizing antibodies after three immunizations (Fig. 5D). The dnsp16 mutant strain had a 25% mortality rate at the high dose, while 100% of the cats immunized at the low dose survived (Fig. 5C). The dnsp16 mutant strain induced the production of lower levels of neutralizing antibodies than dnsp14 at both immunization doses, and half of the cats immunized at the low dose did not produce neutralizing antibodies (Fig. 5E). In summary, although the dnsp14 mutant strain induced a strong humoral immune response and the production of high levels of neutralizing antibodies at both the high and low immunization doses, the safety of immunization needs to be improved; the survival rate of adult cats immunized with the dnsp16 mutant strain at low doses was 100%, but this mutant induced a weak humoral immune response and the production of only low levels of neutralizing antibodies at that dose.

### Fifty percent of cats in the dnsp14-immunized group were able to resist challenge by the highly pathogenic strain QS-79

**Fig 6:**
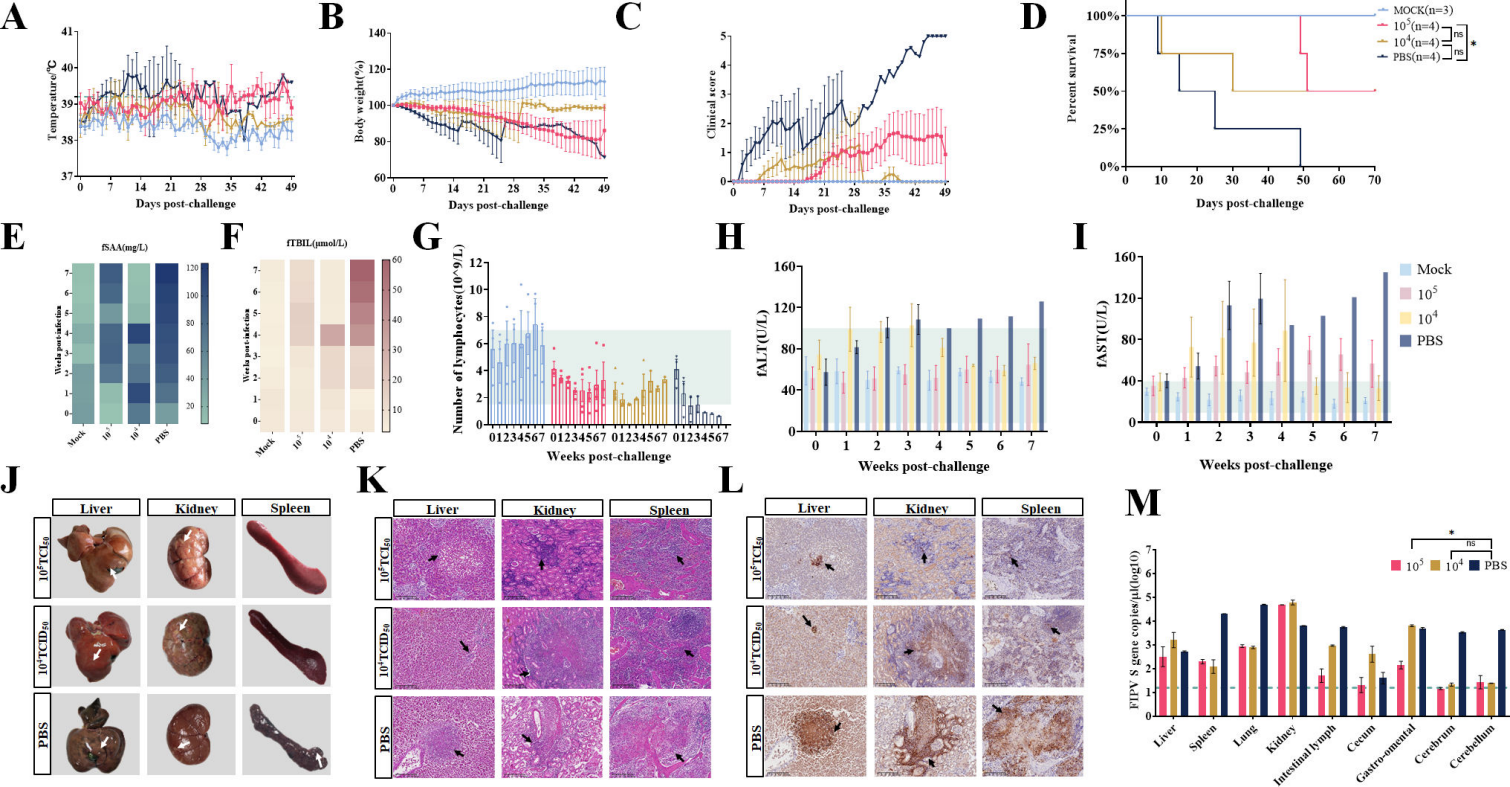
Fifty percent of cats in the dnsp14 immune group were able to resist attack by the highly pathogenic strain QS-79. (**A–D**) Changes in body temperature, body weight, clinical score, and survival rate of the challenge test after dnsp14 immunization in cats. Cats with abnormal clinical status as follows: (A) cats with fever (>39°C); (**B**) cats with anorexia; (**C**) cats with lethargy; (**D**) cats with eye and nose discharge; and (**E**) cats with abnormal excretion. Scoring is based on the accumulation of the above-appearing symptoms, one point for each item. (**E-I**) Biochemical and blood routine analysis of challenge test after dnsp14 immunization in cats. Feline serum amyloid A (fSAA), feline total bilirubin (fTBIL), feline alanine aminotransferase (fALT), and feline aspartate aminotransferase (fAST). (**J–L**) Gross necropsy and histopathological analysis of the challenge test after dnsp14 immunization in cats. After the challenge of the dnsp14 immune group, the liver and kidneys developed purulent granulomatous lesions typical of FIPV (white arrows), H&E staining of various organs showed obvious inflammatory cell infiltration in liver, kidney, and spleen (black arrows), and immunohistochemical detection of these diseased tissues showed obvious FIPV-N antigen signals (black arrows, brown). (**M**) Viral load in tissues and organs of challenge test after dnsp14 immunization in cats. Error bars represent the standard error of the mean (SEM). Statistical significance compared to the high-dose immunization group, the low-dose immunization, and PBS strain at each concentration was determined by one-way ANOVA and indicated with an asterisk (*); *P < 0.05.

On day 80 after the first immunization, all cats were challenged with 1 mL of 10^5.5^ TCID_50_/mL QS-79 for challenge assays (*n* = 4 for each group). The results showed that the cats in the PBS-immunized group, the dnsp14 high-dose immunization group, and the dnsp14 low-dose immunization group exhibited clinical signs of elevated body temperature and weight loss immediately after infection, and the cats in the PBS group had the most severe clinical signs, with all cats exhibiting fever during infection (Fig. 6A and B). The results of clinical scoring showed that 50% of the cats in the PBS group developed secondary bacterial infection in the middle of the infection, and all cats developed severe FIP symptoms of respiratory distress and hind limb paralysis on the eve of death, while animals in the dnsp14-immunized group all developed milder symptoms and did not develop neurological symptoms or respiratory distress. The dnsp14 low-dose and high-dose immunizations delayed the onset of disease in adult cats by 1 week and 2 weeks, respectively (Fig. 6C). All cats in the PBS group died within 49 days of challenge, while 50% of animals in all dnsp14-immunized groups were protected from the highly pathogenic strain QS-79, except one cat that did not produce antibodies in the low-dose group that died on day 10 after challenge, while the other dnsp14-immunized adult cats exhibited prolonged survival (Fig. 6D). Routine blood and biochemical analyses showed that all cats immunized with the dnsp14 mutant strain exhibited a transient decline in lymphocyte levels after the challenge, and fSAA levels gradually increased after the challenge, with those of the low-dose immunized group recovering more quickly after the infection (Fig. 6E). The lymphocyte count continued to decrease after the challenge, and fTBIL, fALT, and fAST levels continued to increase after the challenge, with fAST levels well above the normal range by the end of the first week of infection (Fig. 6F through I). The fALT and fAST levels were elevated in the dnsp14-immunized group cats for 3–4 weeks after the challenge and then gradually returned to normal levels. The biochemical and routine blood test abnormalities in the PBS group were irreversible. Pathological analyses and viral load measurements were performed on cats in the experimental group. Autopsy showed the typical granulomatous lesions of FIPV infection in the kidney (shown by white arrows) after the dnsp14-immune group cats were challenged with the virus, while the lesions in other organs were less severe than those in the PBS-immunized cats (Fig. 6J). By H&E staining, significant inflammatory cell infiltration was observed in the liver, kidney, and spleen of cats in all three experimental groups. The dnsp14 high-dose immunization group cats did not show significant FIP lesion features in the liver after the challenge, but H&E staining showed hepatocyte swelling and cytoplasmic vacuolization in the liver tissue (Fig. 6K). The immunohistochemistry results showed that the pathological damage in the liver, kidney, and spleen in all three experimental groups was caused by FIPV infection (Fig. 6L). Viral loads in organs were significantly elevated in the dnsp14 high-dose immunized challenge group and the PBS-immunized challenge group. The dnsp14 high-dose immunized challenge group had viral loads below the limit of detection in the cecum, brain, and cerebellum and lower viral loads in the liver, spleen, lungs, and lymph nodes (Fig. 6M). The above data indicate that the dnsp14 mutant strain can induce high levels of neutralizing antibodies and provide partial protection for adult cats. Administration of the dnsp14 mutant strain at different immunization doses provided consistent protection in adult cats, with 50% of adult cats protected against the highly pathogenic strain QS-79, and 50% of cats that died had remission of clinical manifestations and pathological damage caused by FIPV infection and a prolonged survival period.

### None of the different doses of dnsp16 protected against the FIPV QS-79 challenge

**Fig 7:**
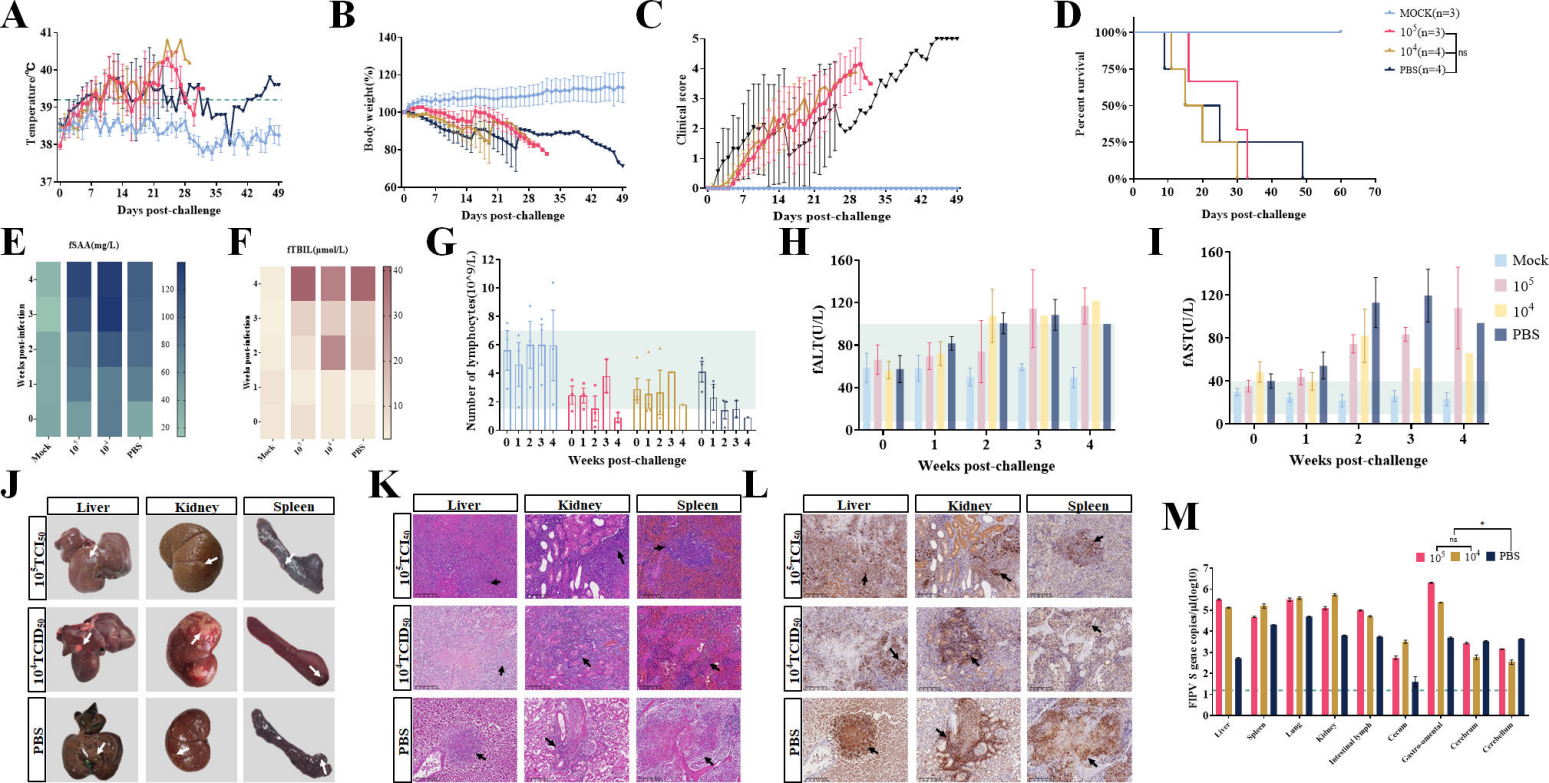
None of the different doses of dnsp16 protects against FIPV attacks. (**A–D**) Changes in body temperature, body weight, clinical score, and survival rate of the challenge test after dnsp16 immunization in cats. Cats with abnormal clinical status as follows: (A) cats with fever (>39°C); (**B**) cats with anorexia; (**C**) cats with lethargy; (**D**) cats with eye and nose discharge; and (**E**) cats with abnormal excretion. Scoring is based on the accumulation of the above-appearing symptoms, one point for each item. (**E–I**) Biochemical and blood routine analyses of the challenge test after dnsp16 immunization in cats. Feline serum amyloid A (fSAA), feline total bilirubin (fTBIL), feline alanine aminotransferase (fALT), and feline aspartate aminotransferase (fAST). (**J-L**) Gross necropsy and histopathological analysis of the challenge test after dnsp16 immunization in cats. After the challenge of the dnsp16 immune group, the liver and kidneys developed purulent granulomatous lesions typical of FIPV (white arrows), H&E staining of various organs showed obvious inflammatory cell infiltration in liver, kidney, and spleen (black arrows), and immunohistochemical detection of these diseased tissues showed obvious FIPV-N antigen signals (black arrows, brown). (**M**) Viral load in tissues and organs of the challenge test after dnsp16 immunization in cats. (Postscript: PBS group data shown in Fig. 6 and 7 were from the same batch of experiment.) Error bars represent the standard error of the mean (SEM). Statistical significance compared to the high-dose immunization group, the low-dose immunization, and PBS strain at each concentration was determined by one-way ANOVA and indicated with an asterisk (*); *P < 0.05.

On day 80 after the first immunization, all cats were challenged with 1 mL of 10^5.5^ TCID_50_/mL QS-79 for challenge assays. The results showed that dnsp16 immunization at different doses induced elevated body temperature immediately after injection and persistent fever, and the body weight of the cats dropped to 80% of their initial body weight within 1 month (Fig. 7A and B). Clinical symptoms such as loss of appetite, depression, dyspnea, and ataxia were not significantly different from those in the PBS-immunized group, and the clinical score continued to increase through 1 month (Fig. 7C). Cats in both the high-dose immunization group and the low-dose immunization group died of natural causes within 30 days (Fig. 7D). There was no significant difference in mortality or time to death among the three experimental groups. Cats in all three experimental groups showed a decline in lymphocyte levels after the challenge, with the cats in the two dnsp16 immunization groups showing a slower decline than those in the PBS group. And fSAA, fTBIL, fALT, and fAST levels continued to increase after infection with QS-79 (Fig. 7E through I). Pathological autopsy of the deceased cats showed that the organ lesions caused by the post-immunization challenge with dnsp16 were highly pronounced, with typical FIP granulomatous lesions in the liver, kidney, and spleen (Fig. 7J). The liver and kidney lesions were most pronounced in the challenge group after immunization with low-dose dnsp16, and H&E staining of the lesion sites showed severe pathological lesions in all tissues. The granulomas were centered on necrotic tissue with massive inflammatory infiltration in the surrounding connective tissue (Fig. 7K). In addition, immunohistochemistry was performed on the lesion sites, and all sections showed strong positive signals (Fig. 7L). Viral load assessment showed that adult cats immunized with the dnsp16 mutant strain had higher levels of virus in the liver, spleen, lung, kidney, lymph nodes, cecum, and greater omentum than those in the PBS-immunized group (Fig. 7M). The results of the challenge protection experiment with different dnsp16 immunization doses indicate that low levels of neutralizing antibodies are unable to block FIPV infection. Higher clinical scores, more pronounced organ lesions, and higher viral loads after the challenge suggest that dnsp16 vaccination may induce enhanced disease in adult cats.

## DISCUSSION

Although FIPV pathogenesis is immunologically complex and the molecular mechanisms involved have not been fully elucidated, there is no doubt that FIPV attacks the immune system and thus has a negative impact on the host (29). The spike gene is crucial for FIPV, and some research has proved that the spike gene correlates with FIPV pathogenesis in many instances (30, 31). Yet previously, a QS-79 recombinant virus obtained by replacing the S gene of the type II nonpathogenic strain 79-1146 with that of the type I QS strain was highly virulent in adult cats, thus demonstrating that the Spike gene is not the key pathogenic gene of FIPV in some cases (28). The MTase activity of nsp14 and nsp16 has been shown to correlate with virulence in various other types of coronaviruses and is essential for viral replication and host immune response evasion. Therefore, in this study, the FIPV nonstructural proteins nsp14 and nsp16 were selected as virulence targets to identify their active sites for the development of live attenuated vaccines.

The MTase functions of nsp14 and nsp16 have been demonstrated in various other coronaviruses, and viruses deficient in enzymatic activity have reduced pathogenicity both *in vitro* and *in vivo*. *In vitro* biochemical assays and complementary assays of yeast N7-MTase activity showed that the N7-MTase activity of the above mutant proteins was deficient (32). Initial attempts to recover mutants in nsp14 and nsp16 that have been reported to have significantly reduced enzyme activity failed to achieve the desired results, including those targeting N379, Y414, D330, and D350 single-site mutations in the N7-MTase structural domain of nsp14, P334-K335 double-site mutations, H288 single-site mutations in the ExoN structural domain, and nsp16 K-D-K-E conserved motif tetra mutation. It is thus suggested that the above loci are required for FIPV replication at the cellular level. Therefore, in this study, we identified and rescued mutants with alterations of the above sites in FIPV by sequence alignment and found that only FIPV QS-79 strain viruses with the mutation in N415A (corresponding to MHV N416A) could be rescued. The nsp16 D129 site is a weakly conserved site for coronaviruses, and FIPV strains with D129 mutations are also easily rescued (23). Accordingly, the FIPV nsp16 D129A mutant strain was also easily rescued. Moreover, attempts to mutate both the nsp14 N415 locus and the nsp16 D129 locus revealed that they could not be recovered in cells. This may be because the virulent strain with nsp14 single mutation still maintained partial MTase function and was able to maintain proliferation capacity in the cells. Similarly, the nsp16 single-mutant strain cap-0 is structurally intact, and the viral translation function and evasion of host immune surveillance remain partially normal, allowing the mutant to retain its proliferative capacity in cells. After the mutation sites were determined, nsp14, nsp16, nsp14 N415A, and nsp16 D129A mutation proteins were expressed to measure the MTase activity, and it turns out that N415 and D129 are important MTase activity sites of nsp14 and nsp16, respectively. When both nsp14 and nsp16 MTase activities are absent, the production of the RNA cap is severely impaired, so the transcript of the resultant strain is unable to be translated in the cell, and the host sensor RIG-I is more likely to recognize non-self RNA to induce antiviral clearance (33).

Although numerous studies have shown that mutations at the abovementioned loci in other coronaviruses lead to attenuation of viral replication, the FIPV nsp14 N415A and nsp16 D129A mutant strains do not have reduced replication capacity at the cellular level but do induce an increase in ISG, IFN, and TNF expression compared to that by wild-type strains, activating host innate immunity. The detailed mechanisms responsible for this phenotype need to be investigated in depth and can be further explored to determine whether other mechanisms of FIPV immune escape counteract the effect of RNA cap demethylation on viral replication in cells (34). *In vivo* infection experiments showed reduced mortality and relatively mild clinical signs in adult cats infected with dnsp14 and dnsp16. The dnsp14 did not decrease in pathogenicity with dose, induced high levels of neutralizing antibodies similar to natural infection at both immunization doses, and provided 50% protection against lethal challenge by a potent strain. By contrast, dnsp16 was not pathogenic at the lower immunization dose of 10^4^ TCID_50_, induced lower levels of neutralizing antibodies, and did not protect against highly virulent challenge. The nsp16 structural determination demonstrated that D129 is a key site for 2′-O-MTase activity, and *in vitro* biochemistry assays showed that mutation of FCoV nsp16 D129 to alanine decreased the enzymatic activity of nsp16 by more than 95% (35). Therefore, during *in vivo* infection, viruses lacking the cap-1 structure are easily cleared by the body’s immune system, especially via the host sensor MDA5 (20). In adult cats infected with dnsp16, viral particles were detected only in the lungs and spleen, and the viral load was significantly lower than that in the dnsp14-infected group cats, suggesting that the dnsp16 mutant strain has limited replication *in vivo* and does not effectively activate the adaptive immune response. The nsp14 N415 site does not directly affect SAM binding but rather acts as a consolidator and stabilizer of the methyl receptor, and the effect of mutations at this site on N7-MTase activity has not been biochemically characterized (36). The viral detection in multiple organs after dnsp14 infection *in vivo* suggested that the nsp14 N415A mutation may result in partial loss of function, allowing the virus to evade host immune surveillance and disseminate systemically. Therefore, the humoral immune response to dnsp14 is stronger than that to dnsp16.

The two mutant strains, dnsp14 and dnsp16, induce very different protective effects in adult cats. A major obstacle to previous attempts at FIPV vaccine development was that neutralizing antibodies did not protect but rather accelerated the disease process and enhanced clinical manifestations (27). In the presence of subneutralizing levels of antibodies or nonneutralizing antibodies, mononuclear macrophages enhance viral uptake via anti-S antibodies through Fc receptors, leading to antibody-dependent enhancement (ADE) (37, 38). The dnsp14 mutant virus induced the production of neutralizing antibodies against S proteins but did not cause ADE after challenge due to high antibody levels. By contrast, adult cats immunized with dnsp16 were unprotected, and the marked enhancement of some of the clinical manifestations and pathological changes suggested that an ADE effect may have occurred. The dnsp14 challenge protection experiments demonstrated that high levels of neutralizing antibody titers helped adult cats resist virulent FIPV challenge. The failure of protection in a subset of cats implied that the adaptive immune response generated by the weakly virulent vaccine alone was not sufficient to provide 100% protection. This result is common in vaccine development for FIPV, as researchers have similarly elicited only partial protection when cats were vaccinated with nonvirulent live virus or sublethal doses of virus (25). In contrast to the 50% survival rate of adult cats with dnsp14 10^5^ TCID_50_ immunization doses for the first challenge, we performed a second challenge in cats surviving from the first challenge, which resulted in 100% protection (Fig. S2). Neutralizing antibody levels changed only slightly during this period, probably because the challenge with the virulent strain QS-79 further enhanced cellular immunity on top of the immunity induced by the mutant strain, leading to an increased protective effect against the second challenge. Therefore, induction of T cell-mediated potent cellular immunity is required in the design of live attenuated FIPV vaccines to confer improved protection in cats (39). In addition, regarding the residual pathogenicity of dnsp14, mutation of other virulence genes can be considered for introduction into the vaccine design platform. It has been shown that adjuvant proteins are usually associated with viral virulence (40, 41). The mutants lacking the type II FIPV ORF3 gene or ORF7 gene can proliferate *in vitro* and display an attenuated phenotype in cats, resulting in the induction of high titers of neutralizing antibodies and protecting against lethal challenge (42, 43). Therefore, in dnsp14 mutant strains, virulence may be further attenuated, and protection may be improved by targeted deletion of non-replication-essential accessory genes.

Overall, we successfully constructed and rescued FIPV strains with mutations in the nsp14 N7-MTase active site (N415) and nsp16 2′-O-MTase active site (D129). The mutant strains dnsp14 and dnsp16 were less pathogenic in adult cats, with dnsp16 being more labile. dnsp14 induced the production of high levels of neutralizing antibodies after immunization and provided 50% protection against a virulent FIPV challenge, while dnsp16 induced the production of only low levels of neutralizing antibodies after immunization and did not protect against a FIPV challenge.

## MATERIALS AND METHODS

### Cells and viruses

CRFK cells and HEK293T cells (American Type Culture Collection, ATCC) were grown in Dulbecco’s modified Eagle medium (DMEM; Gibco, Thermo Fisher Scientific, Waltham, MA, USA) containing 10% fetal bovine serum (FBS). Fcwf-4 cells (ATCC) were grown in Eagle’s minimum essential medium (EMEM; ATCC, catalog No. 302003) containing 10% FBS. All cells were incubated at 37°C in a 5% CO_2_ humidified cabinet.

The FIPV strain rQS-79 was stored at −80℃ by our laboratory (28).

### Sequence alignment, homology modeling, molecular simulation, and molecular docking techniques

The amino acid sequences of FIPV 79-1146 (accession OQ311323.1), PEDV CV777 (accession AF353511.1), MHV A59 (accession MF618252.1), TGEV WH-1 (accession HQ462571.1), SARS-CoV-2 Wuhan-Hu-1 (accession NC_045512.2), SARS-CoV Tor2 (accession NC_004718.3), and MERS-CoV EMC (accession NC_ 019843.3) were downloaded from the NCBI database and analyzed by the Clustal Omega and ESPript 3.0 online programs. The amino acid sequences of nsp14 and nsp16 were compared with those of the FIPV QS-79 strain.

Structural simulations of the FIPV QS-79, QS-79 nsp14 N415A, and QS-79 nsp16 D129A mutant strains were performed via the AlphaFold2 online website using the simulated structural model as the receptor structure and SAM as the model substrate to dock the receptor to the ligand using the Auto Dock Vina module for the wild-type and mutant strains, revealing the SAM-binding pocket. If there is no special reminder, QS-79 nsp14 N415A is abbreviated as dnsp14, QS-79 nsp16 D129A as dnsp16.

### N7- and 2′-O-MTase assays

The enzymatic activities of FIPV N7-methyltransferase (nsp14) and 2′-O-methyltransferase (nsp16/nsp10 complex) were evaluated as previously described (18, 44, 45). Recombinant FIPV nsp14 (wild type and N415A mutant), nsp16 (wild type and D129A mutant), and nsp10 were individually expressed in *Escherichia coli* and purified.

Synthetic RNA substrates were prepared *in vitro* and enzymatically capped with either GpppA or m⁷GpppA cap analogs, depending on the specific assay. For the 2′-O-MTase assays, the reaction mixture contained purified nsp16/nsp10 complex, m⁷GpppA-RNA substrate, and [³H]-S-adenosylmethionine ([³H]-SAM) as the methyl donor. The incorporation of the radioactive methyl group onto the RNA was measured using a Wallac 1450 MicroBeta Trilux liquid scintillation counter. The N7-MTase assay was performed similarly, using nsp14 and GpppA-RNA as the substrate.

### Cloning, recovery, and verification of mutant viruses

Using the BAC-FIPV QS-79 infectious cloning platform in our laboratory, sgRNA primers and recombinant primers were designed to mutate the nsp14 and nsp16 enzymatic activity-related sites of the QS-79 strain in combination with CRISPR-Cas9 technology (detailed sgRNA sequences are shown in Table 1, mutant primer sequences were shown in Table 2). After fusing the forward and reverse primers into double-stranded DNA, T7 RNA transcription was performed to obtain sgRNA, followed by cas9 enzyme digestion of the BAC FIPV plasmid, recovery, and PCR amplification of the nsp14 and nsp16 genes and mutants.

**TABLE 1:** Sequences of the primers used for CRISPR-Cas9

Primer	Sequence
sgRNA	AAAAGCACCGACTCGGTGCCACTTTTTCAAGTTGATAACGGACTAGCCTTATTTTAACTTGCTATTTCTAGCTCTAAAAC
sg-nsp14 F	GATCACTAATACGACTCACTATAGATAATTTTAAGACAAGTGAGTTTTAGAGCTAGAAA
sg-nsp14 R	GATCACTAATACGACTCACTATAGCACAAGTGAAGTTACCTGAGTTTTAGAGCTAGAAA
sg-nsp16 F	GATCACTAATACGACTCACTATAGTAGCTGATAAGATCATGGTGGTTTTAGAGCTAGAAA
sg-nsp16 R	GATCACTAATACGACTCACTATAGTTGTTGGATTGCTAAGGAAGTTTTAGAGCTAGAAA

**TABLE 2:** Prokaryotic expression of nsp14 and nsp16 mutant primers

Primer	Sequence
nsp14-N415A F	GCATTGTATGTTGCTAATCATGCCT
nsp14-N415A R	AGGCATGATTAGCAACATACAATGC
nsp14 F	TCTGATAATTTTAAGACAAGTGACGGC
nsp14 R	CAACGACATTAGTAGTAATGCCGTCA
nsp16-D129A F	TTTACTTATATCTGCTTTATATGATGGCTC
nsp16-D129A R	GAGCCATCATATAAAGCAGATATAAGTAAA
nsp16 F	GCTGTTGTAGCTGATAAGATCATGGTGAG
nsp16 R	ATTTCTAATGAGTAACTTACCCTTCCTTAGCAA

The recovered vectors and mutant fragments were reconstituted and inserted into DH10B receptor cells via transformation. Positive colonies were selected and sent for sequencing analysis. BAC plasmid extraction was performed for sequencing-positive bacteria, and the plasmids were transfected into HEK-293T cells grown to 90%–100% confluence. After transfection for more than 24 h, the supernatants were inoculated into CRFK cells. When obvious cytopathic lesions appear, the virus suspension was collected and stored at −80°C.

### Viral growth kinetics

CRFK and Fcwf-4 cells were seeded in 24-well plates and then inoculated with QS-79, dnsp14, or dnsp16 at an MOI of 0.01 in the presence of inhibitor. After 1 h of incubation, the cells were washed twice with PBS, followed by the addition of maintenance medium, and the supernatants of the cells were collected. The viral titers were determined by a TCID_50_ assay. Briefly, CRFK cells were prepared at 100% confluence in 96-well plates and were washed three times. Serial 10-fold dilutions of virus samples were inoculated in eight replicates per dilution. The cells were monitored for 3–5 days, and virus titers were calculated by using the Reed-Muench method.

### Virus plaque assay

Fcwf-4 cells were inoculated in six-well plates and placed in a 37°C, 5% CO_2_ cell incubator to grow a full monolayer. The virus samples to be tested were diluted 10-fold in sterile 1.5 mL EP tubes. The cell culture supernatant was aspirated from the six-well plate, the cells were washed three times with PBS, and the virus solution diluted with cell growth solution was added and incubated for 2 h at 37°C in a 5% CO_2_ cell incubator. DMEM cell maintenance solution (2×) and 2% low-melting-point agarose were mixed 1:1 to make a plaque assay solution, the virus solution was aspirated from the six-well plate, and the cells were washed twice with sterile PBS. The six-well plates were placed in a 4°C refrigerator to condense (10–15 min) and then incubated in a 37°C, 5% CO_2_ cell culture incubator for 2–3 d. After the plaques emerged, the plates were fixed with 4% paraformaldehyde at 37°C for 15 min, 2 mL of PBS was added to remove the agarose cover layer, and 1% crystal violet was added to completely cover the cells and stain them for 1 h. Finally, the cells were washed with PBS to remove the crystal violet, dried, and photographed to observe the morphology of virus plaques.

### Indirect immunofluorescence

CRFK cells were spread onto six-well plates at 80% cell density and inoculated with 0.01 MOI of virus, the medium was discarded when 60% lesions appeared, and the cells were fixed with 4% paraformaldehyde for 30 min. The cells were permeabilized with 0.1% Triton X-100, blocked with 2% BSA for 30 min, incubated with primary antibody (1:1,000 dilution) for 1 h, washed three times with PBS, incubated for 1 h with fluorescent secondary antibody (1:1,000 dilution), and washed three times with PBS. DAPI was added, and the cells were washed three times with PBS after 5 min. Then, the cells were observed and imaged with a fluorescence microscope, and images were acquired accordingly.

### Western blotting

CRFK cells were seeded in 12-well plates at a density of 1 × 10^6^ cells per well. When the cells had grown to 80%–90% confluence, the virus was added at an MOI of 0.01. After incubation at 37°C for 36 h, the cells were lysed on ice in lysis buffer H containing protease inhibitors. The supernatants were used for Western blot assays. After SDS-PAGE, the proteins were transferred to polyvinylidene difluoride (PVDF) membranes (Bio-Rad, Hercules, CA, USA). After being blocked with 5% skim milk at room temperature for 3 h, the membranes were incubated with an anti-FIPV NP (nucleocapsid protein) polyclonal antibody and an anti-GAPDH monoclonal antibody for 2 h. Then, the membranes were incubated with an HRP-conjugated secondary antibody. The membranes were visualized using an enhanced chemiluminescence system (Amersham Imager 600, GE Healthcare, Pittsburgh, PA, USA) for 40 min.

### SYBR green real-time fluorescence quantitative PCR

Fcwf-4 cells were inoculated into a six-well plate, infected at an 80% density at an MOI of 0.01, and incubated for 24 h. Then, the cell culture medium was removed, the cells were washed twice with sterile PBS, RNA was extracted from the cells by the TRIzol method, 1 µg of total RNA sample was removed, and cDNA was obtained according to the instructions of the Vazyme reverse transcription kit. The reverse-transcribed cDNA samples were amplified with SYBR qPCR Master Mix in an ABIViiA7 fluorescence quantification instrument, and different primers were used to detect the differences in the expression of specific genes in different samples. The amplification conditions were 95°C for 3 min for predenaturation, followed by 95°C for 15 s and 56°C for 30 s for 40 cycles and collection of fluorescent signals. The primers used to detect gene expression are shown in Table 3.

**TABLE 3:** Specific gene relative expression-level primers

Primer	Sequence
TNF-α F	GACAAGCCAGTAGCCCATGT
TNF-α R	TTGGTCTGGTAGGAAACGGC
ISG15 F	TCCTGGTGAGGAACCACAAGGG
ISG15 R	TTCAGCCAGAACAGGTCGTC
IFNβ F	GAAGGAGGAAGCCATATTGGT
IFNβ R	CTCCATGATTTCCTCCAGGAT
IFITM1-F	AGCTCCCTATCAGGGTCC
IFITM1-R	CTCCAGCTTTGGAATGAGCAGC

### Pathogenicity of mutant viruses in adult cats

Sixteen adult cats were randomly divided into four groups: four in the negative control group (mock), four in the dnsp14 group, four in the dnsp16 group, and four in the QS-79 group (WT). The cats in the three groups were placed in three completely isolated rooms, one cat cage for each cat, to prevent fights and injuries between cats (while avoiding direct contact); each group was dedicated to a specific group during feeding to prevent cross-infection. Cat food and water were provided daily during the experiment (the food basin was washed once per day), and the litter box was cleaned daily. Each cat was analyzed by RT-PCR for pathogenic infections (negative cats could be used for downstream experiments), including those by FCoV, FIV, FHV, FPV, FeLV, and SARS-CoV-2, and the weight and mental status of each cat were recorded before the challenge. QS-79, dnsp14, and dnsp16 were administered orally to the cats in the WT, dnsp14, and dnsp16 groups, respectively. The mental status of each cat was scored daily. At the appropriate time (i.e., when the last cat in the WT group died), the mock control cats were treated with the appropriate volume of PBS.

The number of individuals euthanized for use as a control was measured. The weight of each cat was recorded before autopsy. Gross observations were made during necropsy, and tissues such as the intact lungs, kidneys, liver, spleen, and large intestine of the cats were collected for subsequent viral load testing. The freshly dissected animal tissues were fixed in paraformaldehyde, embedded in conventional paraffin, cut into 4 µm slices for H&E staining and immunohistochemical detection, and finally observed under a light microscope for histopathological changes, which were recorded.

### Immunoprotection against mutant viruses in adult cats

Different immunization gradients were set for 10^5^ TCID_50_ and 10^4^ TCID_50_. One milliliter of 10^5^ TCID_50_/mL dnsp14 virus in the dnsp14 group (six animals) and 1 mL of 10^5^ TCID_50_/mL dnsp16 virus in the dnsp16 group (four animals) were administered for the 10^5^ TCID_50_ immunization dose. The mock group cats (four animals) were inoculated with an equal volume of sterile PBS. Blood was collected and tested for neutralizing antibody levels in cat serum. Cats in each experimental group received QS-79 orally at 1 mL of 10^5.5^ TCID_50_/mL viral solution per cat on day 80 after the first vaccination. Each cat was scored daily for mental status and other indicators. When the appropriate time point was reached, an appropriate number of cats in each group were euthanized for control purposes. Before the autopsy, the weight of each cat was recorded. Gross observations were made during necropsy, and tissues such as the intact lungs, kidneys, liver, spleen, and large intestine of the cats were collected for subsequent viral load testing. The freshly dissected animal tissues were fixed in paraformaldehyde, embedded in conventional paraffin, cut into 4 µm slices for H&E staining and immunohistochemical detection, and finally observed for histological pathological changes under a light microscope, which were recorded. (According to the recommendations of the Animal Ethics Committee, to reduce the number of experimental cats, we arranged the experiments of two mutant viruses at the same time and set up only one batch of PBS groups. Therefore, PBS data shown in Fig. 6 and 7 remain consistent.)

### Neutralizing antibody assay

Cells for the neutralizing antibody assay were prepared 1 day in advance, and when the cells reached 80%–90% confluence, the medium was used to spread the cells. Inactivated serum (30 min at 56°C) was diluted twofold, and the diluted antibody was mixed with 50 µL containing 100 TCID_50_ of virus and incubated at 37°C for 1 h. The incubated virus and serum mixture was added to a 96-well plate. Afterward, the observation of viral lesions was carried out daily, and when the lesions were very obvious, the calculation of the number of cell wells for cytopathology was performed. The neutralizing antibody titer of each serum sample was then determined.

### Clinical testing

Routine blood cell examination: First, 0.5 mL of blood from the forelimb of the cat was drawn into an anticoagulation tube containing EDTA, and the tube was slowly shaken to mix the blood, which was then added to a five-category automatic blood analyzer for automatic detection.

Cat serum amyloid: After routine blood testing, blood samples were centrifuged at 4,500 r/min for 10 min. Ten microliter of the centrifuged supernatant was mixed with diluent, and 100 µL of the diluted serum was added to the reagent strip and detected automatically with an immunofluorescence detector.

Blood biochemical examination: First, 1 mL of venous blood was drawn into a lithium heparin anticoagulation tube and centrifuged at 4,500 rpm/min for 10 min, and then 100 µL of the upper plasma layer was added to the liver and kidney discs for biochemical tests. Total protein (TP), albumin (ALB), globulin (GLOB), albumin-to-globulin ratio (A/G), total bilirubin (TBIL), alanine aminotransferase (ALT), aspartate aminotransferase (AST), glutathione glutamate ratio (ALT/AST), gamma-glutamyl transferase (GGT), urea nitrogen (BUN), creatinine (CREA), urea nitrogen/creatinine ratio (SCR), and glucose (GLU) were determined.

### Viral load

RNA was extracted from gastrointestinal tissues, and cDNA for qPCR (Vazyme, China) was synthesized using HiScript II Q RT SuperMix according to the manufacturer’s instructions. Chamq SYBR qPCR Master Mix (Vazyme, China) was used to carry out qPCR in an ABI Step One Plus. The sequence of the positive primer FIPV-rQS-79-S-gene was 5′-ACCCACGGACAATGGAACAA-3′, and the sequence of the reverse primer was 5′-TAGCAGTGCTTGAGCGTGAA-3′. The method to establish the plasmid standard curve was as follows: the PCR amplification products produced with the above primers were recovered and cloned into the pMD-18t vector (Takara, Japan) as the standard plasmids for FIPV. A series of 10-fold dilutions ranging from 10 to 1,000,000 ng/µL was prepared as standard samples to plot the standard curve. The threshold value was set to 0.05 in the result analysis. According to the standard curve, the virus concentration in these samples was calculated (displayed as genome copies/µL).

### Statistics and analysis

All statistical analyses were performed by GraphPad Prism 9.2.0 software. Data were evaluated using ANOVA. A *P* value < 0.05 was considered statistically significant (*), a *P* value < 0.01 was considered statistically significant (**), and a *P* value<0.001 was considered highly statistically significant (***).

## Data Availability

The data that support the findings of this ·study are openly available in this article and are available from the corresponding author after request.

## SUPPLEMENTAL MATERIAL

Figure S1 jvi.00839-25-s0001.tif Expression of innate immune genes in cat after virus challenge.

Figure S2 jvi.00839-25-s0002.tif Second challenge of dnsp14 group surviving cat.

Supplemental legends jvi.00839-25-s0003.docx Legends for Fig. S1 and S2.

ASM does not own the copyrights to Supplemental Material that may be linked to, or accessed through, an article. The authors have granted ASM a non-exclusive, world-wide license to publish the Supplemental Material files. Please contact the corresponding author directly for reuse.

## References

[1] Thiel V, Thiel HJ, Tekes G. 2014. Tackling feline infectious peritonitis via reverse genetics. Bioengineered 5:396–400. doi:10.4161/bioe.32133 25482087 PMC4601228

[2] Brown MA. 2011. Genetic determinants of pathogenesis by feline infectious peritonitis virus. Vet Immunol Immunopathol 143:265–268. doi:10.1016/j.vetimm.2011.06.021 21719115 PMC7132420

[3] Chang HW, Egberink HF, Halpin R, Spiro DJ, Rottier PJ. 2012. Spike protein fusion peptide and feline coronavirus virulence. Emerg Infect Dis 18:1089–1095. doi:10.3201/eid1807.120143 22709821 PMC3376813

[4] Barker EN, Tasker S. 2020. Advances in molecular diagnostics and treatment of feline infectious peritonitis. Adv Small Animal Care 1:161–188. doi:10.1016/j.yasa.2020.07.011

[5] Bálint Á, Farsang A, Zádori Z, Hornyák Á, Dencső L, Almazán F, Enjuanes L, Belák S. 2012. Molecular characterization of feline infectious peritonitis virus strain DF-2 and studies of the role of ORF3abc in viral cell tropism. J Virol 86:6258–6267. doi:10.1128/JVI.00189-12 22438554 PMC3372193

[6] Hartmann K. 2005. Feline infectious peritonitis. Vet Clin North Am Small Anim Pract 35:39–79. doi:10.1016/j.cvsm.2004.10.011 15627627 PMC7114919

[7] Pedersen NC, Kim Y, Liu H, Galasiti KA, Eckstrand C, Groutas WC, et al.. 2018. Efficacy of a 3C-like protease inhibitor in treating various forms of acquired feline infectious peritonitis. J Feline Med Surg 20:378–392. doi:10.1177/1098612X17729626 28901812 PMC5871025

[8] Kenney SP, Wang Q, Vlasova A, Jung K, Saif L. 2021. Naturally occurring animal coronaviruses as models for studying highly pathogenic human coronaviral disease. Vet Pathol 58:438–452. doi:10.1177/0300985820980842 33357102

[9] de Barros B de CV, Castro CMO de, Pereira D, Ribeiro LG, Júnior JWBD, Casseb SMM, Holanda GM, Cruz ACR, Júnior ECS, Mascarenhas JDP. 2019. First complete genome sequence of a feline alphacoronavirus 1 strain from Brazil. Microbiol Resour Announc 8. doi:10.1128/MRA.01535-18 PMC6406114 30863824

[10] Brown MA, Troyer JL, Pecon-Slattery J, Roelke ME, O’Brien SJ. 2009. Genetics and pathogenesis of feline infectious peritonitis virus. Emerg Infect Dis 15:1445–1452. doi:10.3201/eid1509.081573 19788813 PMC2819880

[11] Tekes G, Hofmann-Lehmann R, Stallkamp I, Thiel V, Thiel HJ. 2008. Genome organization and reverse genetic analysis of a type I feline coronavirus. J Virol 82:1851–1859. doi:10.1128/JVI.02339-07 18077720 PMC2258703

[12] Terada Y, Kuroda Y, Morikawa S, Matsuura Y, Maeda K, Kamitani W. 2019. Establishment of a virulent full-length cDNA clone for type I feline coronavirus strain C3663. J Virol 93. doi:10.1128/JVI.01208-19 PMC6803248 31375588

[13] Kennedy MA. 2020. Feline infectious peritonitis: update on pathogenesis, diagnostics, and treatment. Vet Clin North Am Small Anim Pract 50:1001–1011. doi:10.1016/j.cvsm.2020.05.002 32563530

[14] Dye C, Siddell SG. 2005. Genomic RNA sequence of Feline coronavirus strain FIPV WSU-79/1146. J Gen Virol 86:2249–2253. doi:10.1099/vir.0.80985-0 16033972 PMC2583351

[15] Yan L, Huang Y, Ge J, Liu Z, Lu P, Huang B, Gao S, Wang J, Tan L, Ye S, Yu F, Lan W, Xu S, Zhou F, Shi L, Guddat LW, Gao Y, Rao Z, Lou Z. 2022. A mechanism for SARS-CoV-2 RNA capping and its inhibition by nucleotide analog inhibitors. Cell 185:4347–4360. doi:10.1016/j.cell.2022.09.037 36335936 PMC9531661

[16] Becares M, Pascual-Iglesias A, Nogales A, Sola I, Enjuanes L, Zuñiga S. 2016. Mutagenesis of coronavirus nsp14 reveals its potential role in modulation of the innate immune response. J Virol 90:5399–5414. doi:10.1128/JVI.03259-15 27009949 PMC4934755

[17] Almazán F, DeDiego ML, Galán C, Escors D, Álvarez E, Ortego J, Sola I, Zuñiga S, Alonso S, Moreno JL, Nogales A, Capiscol C, Enjuanes L. 2006. Construction of a severe acute respiratory syndrome coronavirus infectious cDNA clone and a replicon to study coronavirus RNA synthesis. J Virol 80:10900–10906. doi:10.1128/JVI.00385-06 16928748 PMC1641757

[18] Lu Y, Cai H, Lu M, Ma Y, Li A, Gao Y, Zhou J, Gu H, Li J, Gu J. 2020. Porcine epidemic diarrhea virus deficient in RNA cap guanine-N-7 methylation is attenuated and induces higher type I and III interferon responses. J Virol 94:e00447-20. doi:10.1128/JVI.00447-20 32461321 PMC7394890

[19] Menachery VD, Yount BL Jr, Josset L, Gralinski LE, Scobey T, Agnihothram S, Katze MG, Baric RS. 2014. Attenuation and restoration of severe acute respiratory syndrome coronavirus mutant lacking 2’-o-methyltransferase activity. J Virol 88:4251–4264. doi:10.1128/JVI.03571-13 24478444 PMC3993736

[20] Züst R, Cervantes-Barragan L, Habjan M, Maier R, Neuman BW, Ziebuhr J, Szretter KJ, Baker SC, Barchet W, Diamond MS, Siddell SG, Ludewig B, Thiel V. 2011. Ribose 2’-O-methylation provides a molecular signature for the distinction of self and non-self mRNA dependent on the RNA sensor Mda5. Nat Immunol 12:137–143. doi:10.1038/ni.1979 21217758 PMC3182538

[21] Menachery VD, Debbink K, Baric RS. 2014. Coronavirus non-structural protein 16: evasion, attenuation, and possible treatments. Virus Res 194:191–199. doi:10.1016/j.virusres.2014.09.009 25278144 PMC4260984

[22] Menachery VD, Gralinski LE, Mitchell HD, Dinnon KH 3rd, Leist SR, Yount BL Jr, Graham RL, McAnarney ET, Stratton KG, Cockrell AS, Debbink K, Sims AC, Waters KM, Baric RS. 2017. Middle east respiratory syndrome coronavirus nonstructural protein 16 is necessary for interferon resistance and viral pathogene. mSphere 2:e00346-17. doi:10.1128/mSphere.00346-17 29152578 PMC5687918

[23] Ye Z-W, Ong CP, Tang K, Fan Y, Luo C, Zhou R, Luo P, Cheng Y, Gray VS, Wang P, Chu H, Chan JF-W, To KK-W, Chen H, Chen Z, Yuen K-Y, Ling GS, Yuan S, Jin D-Y. 2022. Intranasal administration of a single dose of a candidate live attenuated vaccine derived from an NSP16-deficient SARS-CoV-2 strain confers sterilizing immunity in animals. Cell Mol Immunol 19:588–601. doi:10.1038/s41423-022-00855-4 35352010 PMC8961489

[24] Hou Y, Ke H, Kim J, Yoo D, Su Y, Boley P, Chepngeno J, Vlasova AN, Saif LJ, Wang Q. 2019. Engineering a live attenuated porcine epidemic diarrhea virus vaccine candidate via inactivation of the viral 2’-o-methyltransferase and the endocytosis signal of the spike protein. J Virol 93:e00406-19. doi:10.1128/JVI.00406-19 31118255 PMC6639265

[25] Pedersen NC, Black JW. 1983. Attempted immunization of cats against feline infectious peritonitis, using avirulent live virus or sublethal amounts of virulent virus. Am J Vet Res 44:229–234. 6299143

[26] Vennema H, de Groot RJ, Harbour DA, Dalderup M, Gruffydd-Jones T, Horzinek MC, Spaan WJ. 1990. Early death after feline infectious peritonitis virus challenge due to recombinant vaccinia virus immunization. J Virol 64:1407–1409. doi:10.1128/jvi.64.3.1407-1409.1990 2154621 PMC249267

[27] Pedersen NC. 2009. A review of feline infectious peritonitis virus infection: 1963-2008. J Feline Med Surg 11:225–258. doi:10.1016/j.jfms.2008.09.008 19254859 PMC7129802

[28] Wang G, Hu G, Liang R, Shi J, Qiu X, Yang Y, Jiao Z, Chen Y, Shen Z, Li M, Shi Y, Mao J, Peng G. 2021. Establishment of full-length cDNA clones and an efficient oral infection model for feline coronavirus in cats. J Virol 95:e0074521. doi:10.1128/JVI.00745-21 34406859 PMC8513462

[29] Malbon AJ, Russo G, Burgener C, Barker EN, Meli ML, Tasker S, Kipar A. 2020. The effect of natural feline coronavirus infection on the host immune response: a whole-transcriptome analysis of the mesenteric lymph nodes in cats with and without feline infectious peritonitis. Pathogens 9:524. doi:10.3390/pathogens9070524 32610501 PMC7400348

[30] Rottier PJM, Nakamura K, Schellen P, Volders H, Haijema BJ. 2005. Acquisition of macrophage tropism during the pathogenesis of feline infectious peritonitis is determined by mutations in the feline coronavirus spike protein. J Virol 79:14122–14130. doi:10.1128/JVI.79.22.14122-14130.2005 16254347 PMC1280227

[31] Jaimes JA, Whittaker GR. 2018. Feline coronavirus: insights into viral pathogenesis based on the spike protein structure and function. Virology (Auckl) 517:108–121. doi:10.1016/j.virol.2017.12.027 PMC7112122 29329682

[32] Pan R, Kindler E, Cao L, Zhou Y, Zhang Z, Liu Q, Ebert N, Züst R, Sun Y, Gorbalenya AE, Perlman S, Thiel V, Chen Y, Guo D. 2022. N7-methylation of the coronavirus RNA cap is required for maximal virulence by preventing innate immune recognition. mBio 13:e0366221. doi:10.1128/mbio.03662-21 35073761 PMC8787479

[33] Goubau D, Schlee M, Deddouche S, Pruijssers AJ, Zillinger T, Goldeck M, Schuberth C, Van der Veen AG, Fujimura T, Rehwinkel J, Iskarpatyoti JA, Barchet W, Ludwig J, Dermody TS, Hartmann G, Reis e Sousa C. 2014. Antiviral immunity via RIG-I-mediated recognition of RNA bearing 5’-diphosphates. Nature 514:372–375. doi:10.1038/nature13590 25119032 PMC4201573

[34] Kiss I, Poland AM, Pedersen NC. 2004. Disease outcome and cytokine responses in cats immunized with an avirulent feline infectious peritonitis virus (FIPV)-UCD1 and challenge-exposed with virulent FIPV-UCD8. J Feline Med Surg 6:89–97. doi:10.1016/j.jfms.2003.08.009 15123153 PMC7128844

[35] Decroly E, Imbert I, Coutard B, Bouvet M, Selisko B, Alvarez K, Gorbalenya AE, Snijder EJ, Canard B. 2008. Coronavirus nonstructural protein 16 is a cap-0 binding enzyme possessing (nucleoside-2’O)-methyltransferase activity. J Virol 82:8071–8084. doi:10.1128/JVI.00407-08 18417574 PMC2519555

[36] Bálint Á, Farsang A, Szeredi L, Zádori Z, Belák S. 2014. Recombinant feline coronaviruses as vaccine candidates confer protection in SPF but not in conventional cats. Vet Microbiol 169:154–162. doi:10.1016/j.vetmic.2013.10.015 24513277 PMC7117248

[37] Weiss RC, Scott FW. 1981. Antibody-mediated enhancement of disease in feline infectious peritonitis: comparisons with dengue hemorrhagic fever. Comp Immunol Microbiol Infect Dis 4:175–189. doi:10.1016/0147-9571(81)90003-5 6754243 PMC7134169

[38] Takano T, Yamada S, Doki T, Hohdatsu T. 2019. Pathogenesis of oral type I feline infectious peritonitis virus (FIPV) infection: antibody-dependent enhancement infection of cats with type I FIPV via the oral route. J Vet Med Sci 81:911–915. doi:10.1292/jvms.18-0702 31019150 PMC6612493

[39] Vermeulen BL, Devriendt B, Olyslaegers DA, Dedeurwaerder A, Desmarets LM, Favoreel HW, Dewerchin HL, Nauwynck HJ. 2013. Suppression of NK cells and regulatory T lymphocytes in cats naturally infected with feline infectious peritonitis virus. Vet Microbiol 164:46–59. doi:10.1016/j.vetmic.2013.01.042 23434014 PMC7117246

[40] Borschensky CM, Reinacher M. 2014. Mutations in the 3c and 7b genes of feline coronavirus in spontaneously affected FIP cats. Res Vet Sci 97:333–340. doi:10.1016/j.rvsc.2014.07.016 25128417 PMC7111757

[41] Bank-Wolf BR, Stallkamp I, Wiese S, Moritz A, Tekes G, Thiel HJ. 2014. Mutations of 3c and spike protein genes correlate with the occurrence of feline infectious peritonitis. Vet Microbiol 173:177–188. doi:10.1016/j.vetmic.2014.07.020 25150756 PMC7117521

[42] Haijema BJ, Volders H, Rottier PJM. 2004. Live, attenuated coronavirus vaccines through the directed deletion of group-specific genes provide protection against feline infectious peritonitis. J Virol 78:3863–3871. doi:10.1128/jvi.78.8.3863-3871.2004 15047802 PMC374255

[43] Dedeurwaerder A, Desmarets LM, Olyslaegers DAJ, Vermeulen BL, Dewerchin HL, Nauwynck HJ. 2013. The role of accessory proteins in the replication of feline infectious peritonitis virus in peripheral blood monocytes. Vet Microbiol 162:447–455. doi:10.1016/j.vetmic.2012.10.032 23182908 PMC7117191

[44] Jin X, Chen Y, Sun Y, Zeng C, Wang Y, Tao J, Wu A, Yu X, Zhang Z, Tian J, Guo D. 2013. Characterization of the guanine-N7 methyltransferase activity of coronavirus nsp14 on nucleotide GTP. Virus Res 176:45–52. doi:10.1016/j.virusres.2013.05.001 23702198 PMC7114466

[45] Wang Y, Sun Y, Wu A, Xu S, Pan R, Zeng C, Jin X, Ge X, Shi Z, Ahola T, Chen Y, Guo D. 2015. Coronavirus nsp10/nsp16 methyltransferase can be targeted by nsp10-derived peptide in vitro and in vivo to reduce replication and pathogenesis. J Virol 89:8416–8427. doi:10.1128/JVI.00948-15 26041293 PMC4524257

