# Supplementary figures and images for: Mutations in feline infectious peritonitis virus nonstructural protein 14/16 methyltransferase attenuate the pathogenicity of the virus in cats
Source: J Virol. 2025 Sep 9;99(10):e00839-25. doi: 10.1128/jvi.00839-25 (PMC12548424; doi:10.1128/jvi.00839-25)

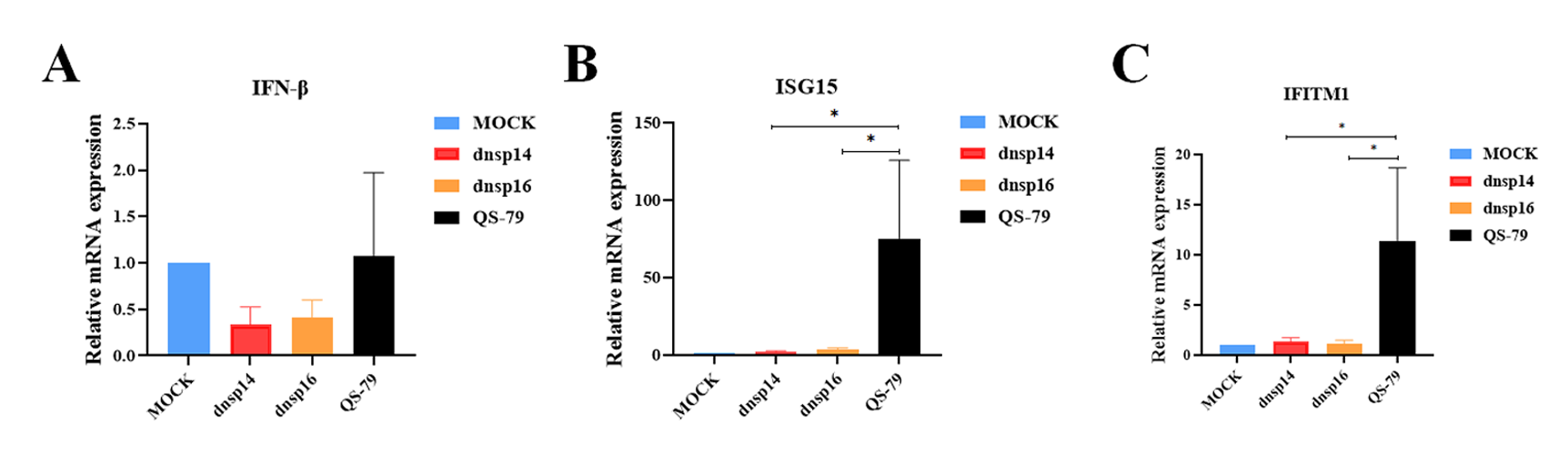

Supplement: Figure S1 — Expression of innate immune genes in cat after virus challenge. [file jvi.00839-25-s0001.tif]

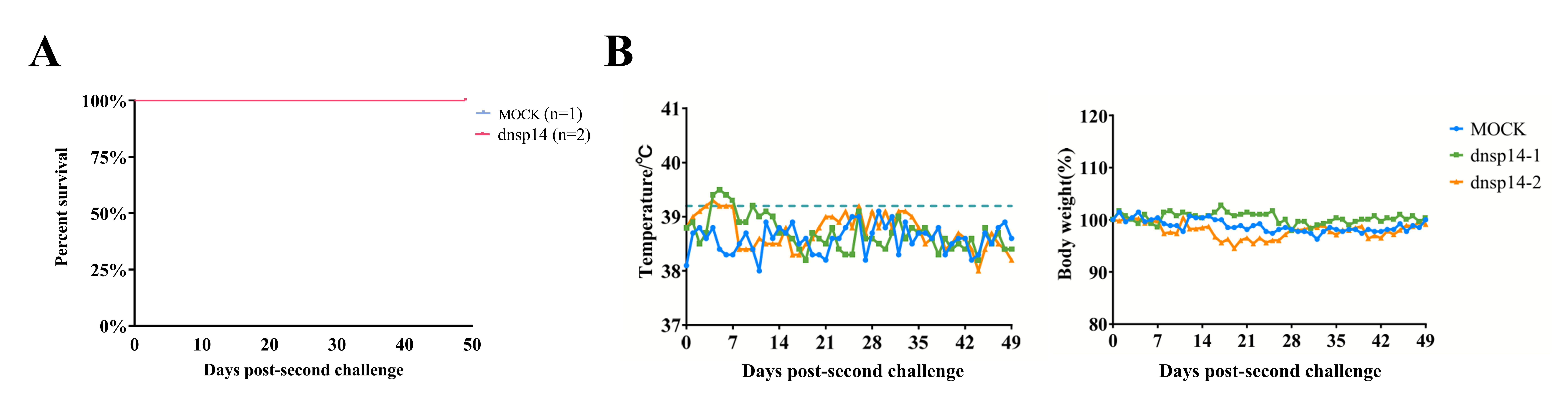

Supplement: Figure S2 — Second challenge of dnsp14 group surviving cat. [file jvi.00839-25-s0002.tif]
