# Supplementary material for: Mutations in feline infectious peritonitis virus nonstructural protein 14/16 methyltransferase attenuate the pathogenicity of the virus in cats
Source: J Virol. 2025 Sep 9;99(10):e00839-25. doi: 10.1128/jvi.00839-25 (PMC12548424; doi:10.1128/jvi.00839-25)
Supplement: Supplemental legends — Legends for Fig. S1 and S2. [file jvi.00839-25-s0003.docx]

**FIG. S1. Expression of innate immune genes in cat after virus challenge.**

(A-C) IFN-β, ISG15 and IFITM1. Error bars represent standard error of the mean (SEM). Statistical significance of each group compared with the QS-79 strain was determined by one-way ANOVA and is indicated by an asterisk (*); *, P < 0.05.

**FIG. S2. Second challenge of dnsp14 group surviving cat.**

(A) Survival rate. (B) Changes of temperature and body weight after re-challenge. Mock: healthy cat.
